# Chemical profiling and quantification of XueBiJing injection, a systematic quality control strategy using UHPLC-Q Exactive hybrid quadrupole-orbitrap high-resolution mass spectrometry

**DOI:** 10.1038/s41598-017-17170-y

**Published:** 2017-12-05

**Authors:** Zhi Sun, Lihua Zuo, Tongwen Sun, Jinfa Tang, Daling Ding, Lin Zhou, Jian Kang, Xiaojian Zhang

**Affiliations:** 1 Department of Pharmacy, The First Affiliated Hospital of Zhengzhou University, No. 1 Jianshe East Road, Zhengzhou, Henan Province 450052 P. R. China; 2 Department of General ICU, The First Affiliated Hospital of Zhengzhou University, No. 1 Jianshe East Road, Zhengzhou, Henan Province 450052 P. R. China; 3 Department of Neurosurgery, The First Affiliated Hospital of Zhengzhou University, No. 1 Jianshe East Road, Zhengzhou, Henan Province 450052 P. R. China; 4 Department of Pharmacy, The First Affiliated Hospital of Henan University of Traditional Chinese Medicine, No. 19 Renmin Road, Zhengzhou, Henan Province 450000 P. R. China

## Abstract

To clarify and quantify the chemical profiling of XueBiJing injection (XBJ) rapidly, a feasible and accurate strategy was developed by applying ultra high performance liquid chromatography-Q Exactive hybrid quadrupole-orbitrap high resolution accurate mass spectrometry (UHPLC-Q-Orbitrap HRMS). A total of 162 components were characterized, including 19 phenanthrenequinones, 33 lactones, 28 flavonoids and 12 phenolic acids and 51 other compounds. Among them, 38 major compounds were unambiguously quantified by comparing with reference standards. Meanwhile, 38 representative compounds were simultaneously detected in XBJ samples by Q-Orbitrap HRMS. Satisfactory linearity and correlation coefficient were achieved with wide linear range. The precisions, repeatability, stability and recovery were meeting requirements. The validated method was successfully applied for simultaneous determination of 38 bioactive compounds in 10 batches XBJ samples. In addition, the similarity evaluation of fingerprintings was applied to assess the quality of XBJ. And the results were evaluated by multiple statistical strategies and five compounds might be the most important chemical markers for chemical quality control of XBJ. Finally, a rapid and simple UPLC-MS/MS method was developed for determination of five markers in XBJ sample. This research established a high sensitive and efficient strategy for integrating quality control, including identification and quantification of XBJ.

## Introduction

XueBiJing injection (XBJ) was comprised of extracts from five Chinese herbals: Carthami Flos, Paeoniae Radix Rubra, Chuanxiong Rhizoma, Salviae miltiorrhizae and Angelicae Sinensis Radix. It has been widely used in China as a blood-activating and anti-endotoxicity drug for the treatment of sepsis and the associated multiple organ dysfunction syndrome (MODS)^1,2^. Modern pharmacological studies indicate that XBJ could protect the endothelium, improve microcirculation, alleviate coagulation and inflammation, and regulate immune response^3,4^. In clinical, XBJ could significantly reduce significantly the value of serum procalcitonin, C-reactive protein and the level of white blood cells in sepsis patients. In addition, the XBJ had an antagonistic effect on inflammatory markers, which could interdict the pathological process of systemic inflammatory response syndrome and reduce the incidence of MODS in order to further improve the prognosis of sepsis patients and reduce the mortality^5,6^. Although XBJ is an effective traditional Chinese medicine (TCM) in treating sepsis, the constituents of which remain largely unknown, and the bioactive components are not completely clear.

According to previous phytochemical and HPLC or UPLC-MS researches, glycosides, flavonoids and phenolic acids were the predominant constituents in XBJ. To date, a few reports have developed a method for qualitative or/and quantitative analysis of compounds in XBJ7–10. Ji *et al*.^7^ established a HPLC method coupled with an ultraviolet detector for the determination of 11 essential compounds in XBJ within 70 min, deficiency existed in terms of analysis time and sensitivity. Huang *et al*.^9^ developed an ultra performance liquid chromatographic (UPLC) method for simultaneous identification and quantification of 13 main components in XBJ and an UPLC/Q-TOF method for identification of 8 major metabolites in XBJ. Huang *et al*.^11^ established an HPLC/DAD/TOF method to identify 23 compounds in XBJ, including amino acids, phenolic acids, flavonoid glycosides, terpene glycosides and phthalides. However, due to the limitation of applied instruments, only high level components were studied in previous studies. To develop a sensitive and accurate method for the comprehensive chemical identification of XBJ, Q-Exactive hybrid quadrupole-orbitrap high-resolution mass spectrometer (Q-Orbitrap HRMS) was employed in the present study.

In this paper, qualitative and quantitative analyses were combined together for the integrated quality control strategy of XBJ. In qualitative analysis, Q orbitrap MS revealed its remarkable high resolution and sensitivity in the chemical identification of XBJ. Q-orbitrap HRMS was employed in the analysis of Chinese medicinal formula for the first time, and it overcame the drawbacks of HPLC and UPLC-MS. In present investigation, 162 unknown compounds were identified, based on their high resolution MS data and the cleavage patterns of 38 reference standards. Meanwhile, in order to avoid the ion response discrimination to different types constituents in XBJ, the fast polarity swinging was realized in one analysis. In addition, the utilization of Q orbitrap HRMS could realize simultaneously qualitative and quantitative determination in one analysis, which shortened analysis time. To the best of our knowledge, this is the first time to report the application of Q-Orbitrap HRMS in simultaneously determining and quantifying so many bioactive constituents in XBJ. The quantitative determination method had been validated and applied for an assay of 10 bathes XBJ samples, and the result could evaluated by fingerprinting and multivariate data analyses (principal component analysis, PCA). Finally, a rapid and simple UPLC-MS/MS method was developed for determination of five markers in XBJ. In one word, we provided a promising and integrated approach for the quality control of XBJ and a solid foundation for the pharmacological and pharmacokinetic study of XBJ.

## Results and Discussions

### Qualitative analysis of XBJ

**Figure 1:**
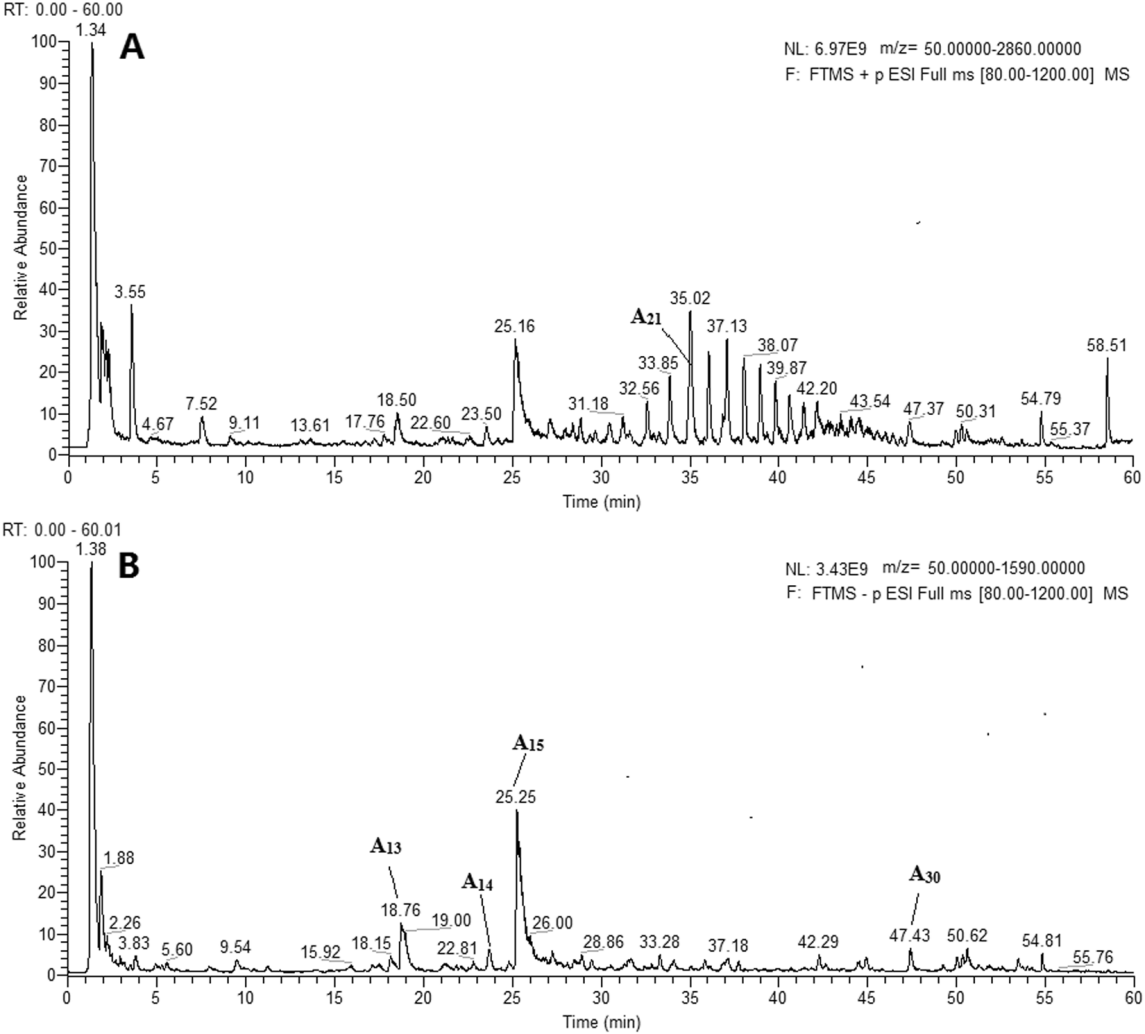
The total ion chromatograms (TIC) of the XBJ sample (**A**) in positive mode (**B**) in negative mode.

**Figure 2:**
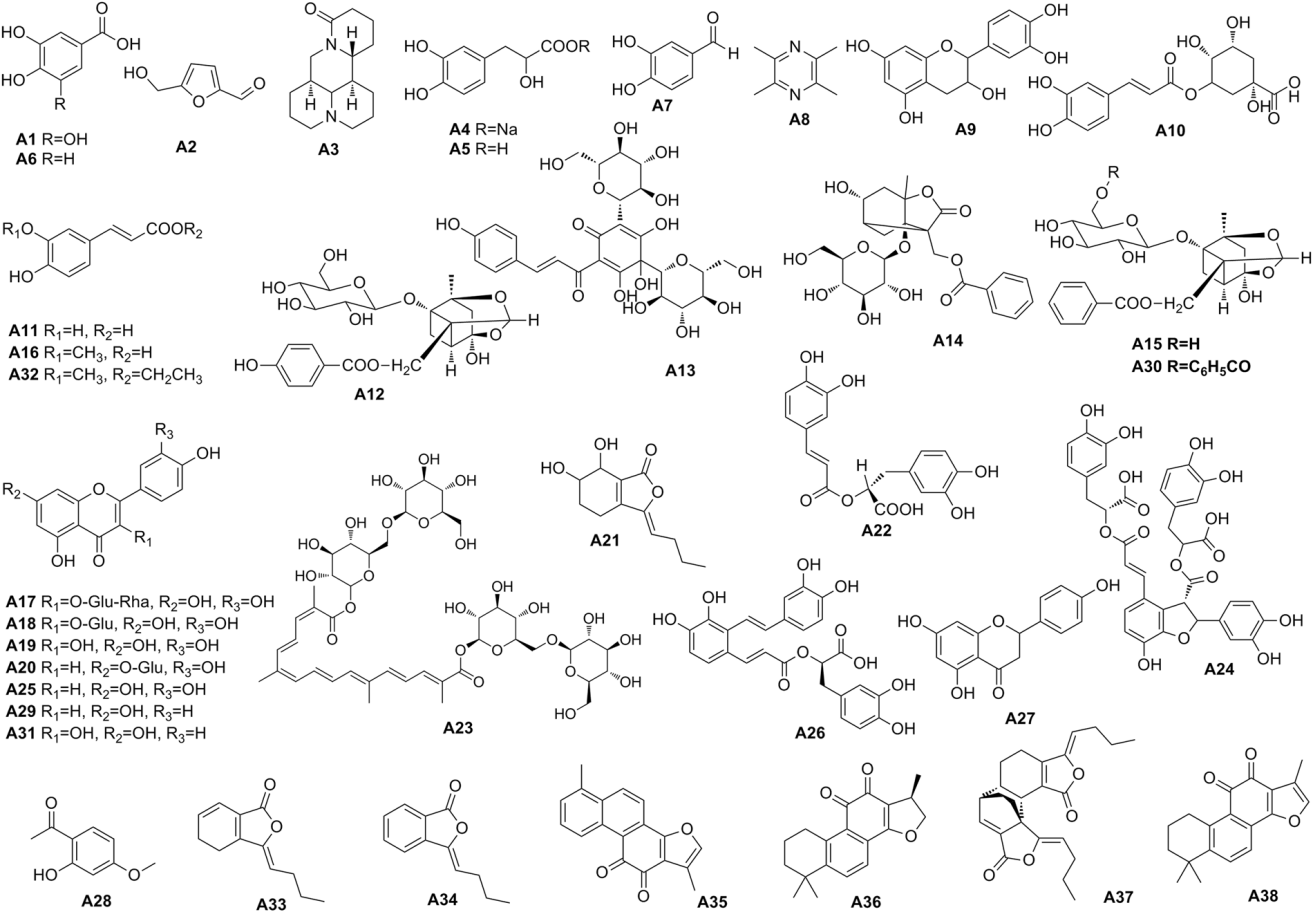
Chemical structures of 38 major components identified from XBJ injection.

A specific UHPLC-Q-Orbitrap HRMS method was developed as a reliable, sensitive and high-throughput method for rapid identification of the components of XBJ regardless of the macro- and micro-constituents. The total ion chromatograms (TIC) of the XBJsample both in positive and negative ion mode are presented in Fig. 1 38 compounds were unambiguously identified based on comparison of retention time and high-resolution accurate mass with that of available reference standards and their chemical structures were shown in Fig. 2. Moreover, the fragmentation patterns and pathways of the standards were investigated in depth to further confirm the structure of their derivatives. For the compounds without available references, the structures were presumed based on the following steps so as to increase the credibility: (1) the molecular formula was established based on high-accuracy protonated precursors such as [M + H]^+^, [M + Na]^+^, [M−H]^−^, or [M + HCOO]^−^ within a mass error of 10 ppm and the fractional isotope abundance; (2) A class of compounds has the same law of cracking, therefore, the standards were utilized to characterize the fragment pathways and diagnostic fragment ions that could be applied for structural elucidation of their derivatives. In addition, some literatures about the compositions of XBJ and five Chinese herbals could be referred. (3) The fragment ions from mass spectrometry were used to further confirm the chemical structure with the aid of Thermo Scientific^TM^ Mass Frontier 7.0^12^.

**Figure 3:**
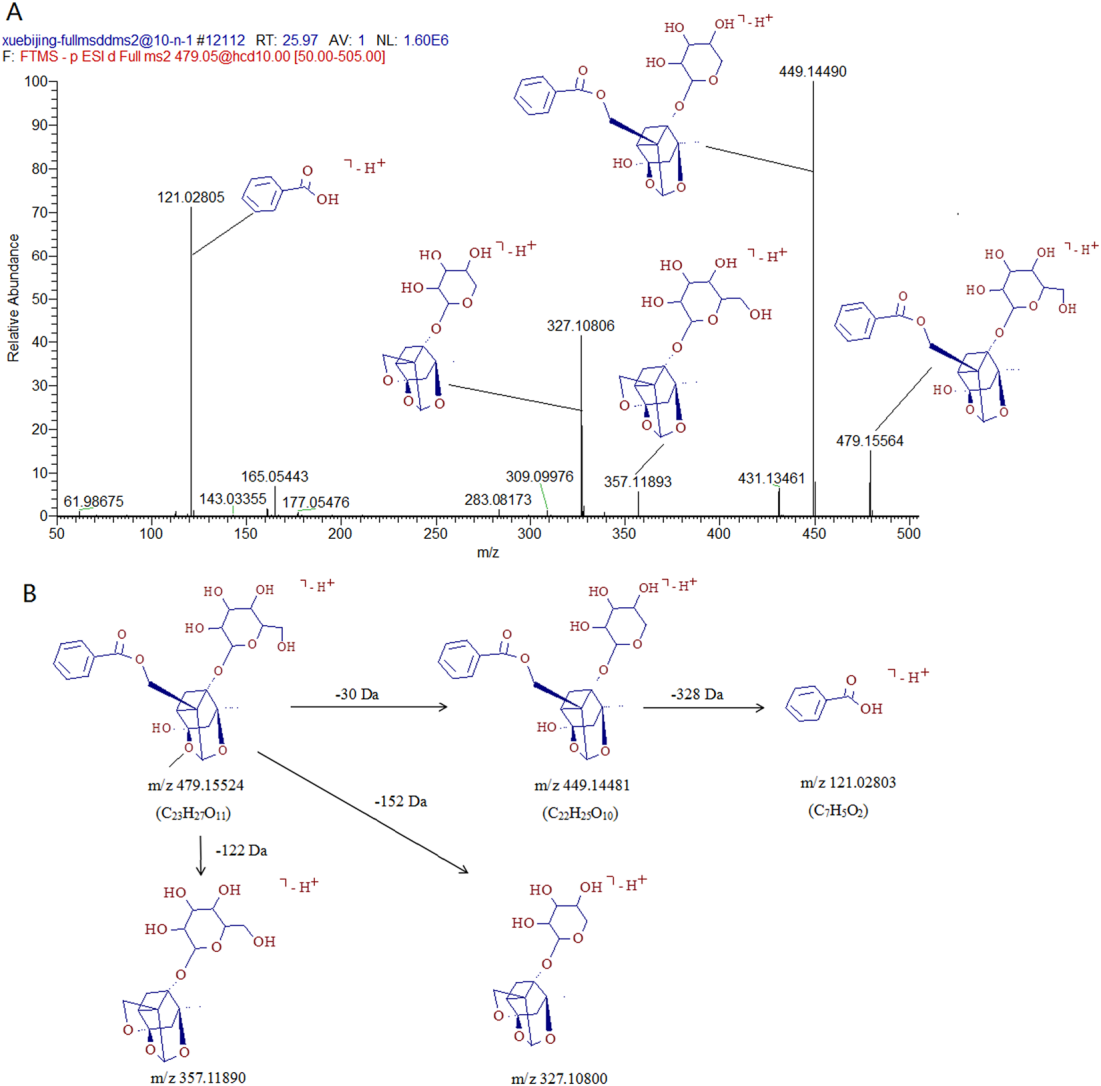
(**A**) The mass spectra and proposed major fragmentation of Paeoniflorin (**B**) Proposed fragmentation pathways of Paeoniflorin.

As for monoterpene glycosides, the loss of CH_3_, H_2_O and CO was observed clearly in their MS/MS spectra. The mass spectra and proposed major fragmentation of representative compounds Paeoniflorin was shown in Fig. 3A and the proposed fragmentation pathways was presented in Fig. 3B. Other constituents were tentatively deduced by the above steps and paeonisuffrone, phenanthrenequinone, senkyunolide, lactones, flavonoids and phenolic compounds dominated the chemical profiling of XBJ^13–23^. Overall, 162 components, including 19 monoterpene glycosides, 19 phenanthrenequinone, 33 lactones, 28 flavonoids and 63 phenolic acid and other compounds, in XBJ were identified or tentatively characterized with their retention times and MS data, which are summarized in Table 1.

**Table 1:** 19 monoterpene glycosides (M), 19 phenanthrenequinone (P), 33 lactones (L), 28 flavonoids (F), 63 phenolic acid and other compounds (O) identified from XBJ by UHPLC-Q-Exactive.

No.	Compounds	*t*_R_ (min)	Formula	Ion mode	ES/expected (m/z)	ES/measured (m/z)	Delta (ppm)	HPLC-ESI-MS/MS (m/z)
M_1_	1-*O*-β-D-glucopyranosyl-paeonisuffr-one	3.21	C_16_H_24_O_9_	−	359.13475	359.13400	−1.853	197.08099, **179.07028**
M_2_	4-O-Methyldesbenzoylpaeoniflorin	8.12	C_17_H_26_O_10_	−	435.15080	435.14944	−3.032	389.14432, **227.09203**
M_3_	Mudanpioside F	9.90	C_16_H_24_O_8_	−	343.13984	343.13879	−3.063	**181.08582**, 151.07512, 109.06439
M_4_	6′-*O*-galloyl-Desbenzoylpaeoniflorin Isomer	12.02	C_23_H_28_O_14_	−	527.14063	527.13922	−2.672	**497.12943**,479.11896,399.09393,313.05627,271.04553(C_11_H_11_O_8_),211.02382,169.01294
M_5_	6′-*O*-galloyl-Desbenzoylpaeoniflorin Isomer	16.34	C_23_H_28_O_14_	−	527.14063	527.13922	−2.672	491.11990,345.11871,313.05621,271.04556, 211.02365(C_9_H_7_O_6_), **169.01297**
M_6_	Oxypaeoniflorin ^a^ (A_12_)	18.15	C_23_H_28_O_12_	−	495.15080	495.14963	−2.362	495.15009,333.09671,281.06604,195.06506, 165.05431, 151.03847, **137.02304**
M_7_	6′-*O*-galloyl-Desbenzoylpaeoniflorin Isomer	20.27	C_23_H_28_O_14_	−	527.14063	527.13947	−2.198	497.13281,399.09381,313.05579,271.04590, 211.02440, **169.01299**
M_8_	Albiflorin ^a^ (A_14_)	23.68	C_23_H_28_O_11_	−	479.15588	479.15445	−2.994	479.11319, 327.10904, **121.02808**
M_9_	Paeoniflorin ^a^ (A_15_)	25.31	C_23_H_28_O_11_	−	479.15588	479.15524	−2.619	449.14481,367.11890,357.18002, **121.02803**
M_10_	Oxypaeoniflorin isomer	26.65	C_23_H_28_O_12_	−	495.15080	495.14890	−1.816	465.13937, 165.05487, **137.02306**
M_11_	Paeoniflorin Isomer	29.44	C_23_H_28_O_11_	−	479.15588	479.15454	−2.807	479., 327.10953, 165.05482, **121.02806**
M_12_	Galloylpaeoniflorin isomer	31.48	C_30_H_32_O_15_	−	631.16684	631.16498	−2.952	631.16595,613.15570,491.11874,399.09253,313.05603,271.04556,211.02396,**169.01303**
M_13_	Paeoniflorin Isomer	31.63	C_23_H_28_O_11_	−	479.15588	479.15414	−2.497	479.15417, 327.11026, 263.07455, 177.05457, 165.05447, 121.02803
M_14_	Galloylpaeoniflorin isomer	32.94	C_30_H_32_O_15_	−	631.16684	631.16516	−2.667	491.12109, 399.09271, 313.05630, 271.04538, 211.02423, **169.01303**, 121.02809
M_15_	Galloylpaeoniflorin isomer	33.44	C_30_H_32_O_15_	−	631.16684	631.16492	−3.047	491.12549, 399.09335, 313.05603, 271.04532, 211.02380, **169.01299**, 121.02781
M_16_	Galloylpaeoniflorin isomer	34.71	C_30_H_32_O_15_	−	631.16684	631.16510	−2.762	431.12598, 313.05792, **169.01309**, 121.02789
M_17_	Benzoyloxypaeoniflorin Isomer	39.58	C_30_H_32_O_13_	−	599.17701	599.17554	−2.460	477.13870,431.13376,281.06613,239.05521, **137.02303**, 121.02802
M_18_	Benzoyloxypaeoniflorin Isomer	41.37	C_30_H_32_O_13_	−	599.17701	599.17566	−2.260	477.14032, 385.09171, 333.09769, 281.06573, 165.05461, **137.02309**, 121.02803
M_19_	Benzoylpaeoniflorin ^a^ (A_30_)	47.43	C_30_H_32_O_12_	−	583.18210	583.18005	−0.953	481.16913,431.13596,165.05434, 135.04375, **121.02801**
P_1_	Tanshinone VI	41.83	C_18_H_16_O_4_	+	297.11213	297.11133	−2.711	279.15570, 261.09055, **184.01868**
P_2_	Cryptotanshinone isomer	50.35	C_19_H_20_O_3_	+	297.14852	297.14746	−3.571	**253.15790**, 238.13448
P_3_	Tanshinone IIB	50.92	C_19_H_20_O_4_	+	313.14344	313.14255	−2.924	313.14240, 295.13196, 269.15262, **251.14232**
P_4_	Tanshinone IIB-isomer	51.09	C_19_H_20_O_4_	+	313.14344	313.14264	−2.541	313.14260, 295.13177, 277.12137, **251.14224**
P_5_	Tanshinone IIB-isomer	52.45	C_19_H_20_O_4_	+	313.14344	313.14149	−3.020	313.14246, **295.13196**, 285.14700, 267.13751
P_6_	Tanshinone V	53.06	C_19_H_22_O_4_	+	315.15909	315.15811	−3.191	315.15793, 297.14737, 279.13748, **267.13718**
P_7_	Tanshinone I isomer	53.47	C_18_H_12_O_3_	+	277.08592	277.08533	−2.457	277.08524, **249.09021**, 178.07695
P_8_	Miltiodiol	53.65	C_19_H_22_O_3_	+	299.16417	299.16432	−2.511	299.16278, 281.15341, **253.15788**
P_9_	Deoxyneocryptotanshinone	53.65	C_19_H_22_O3	+	299.16417	299.16339	−2.611	299.16278, 281.15314, **253.15788**
P_10_	1,2,5,6-tetrahydrotanshinone I	53.72	C_18_H_16_O_3_	+	281.11722	281.11658	−2.280	182.08078, **72.08125**
P_11_	Tanshinaldehyde	54.19	C_19_H_16_O_4_	+	309.11213	309.11136	−2.315	309.11087, 291.09970, **265.12158,223.07478**
P_12_	Tanshinone V-isomer	54.97	C_19_H_22_O_4_	+	315.15909	315.15823	−2.715	297.14822, **253.15796**
P_13_	Cryptotanshinone isomer	54.83	C_19_H_20_O_3_	+	297.14852	297.14792	−0.601	297.14749, **253.15787**
P_14_	Tanshinone IIB-isomer	55.24	C_19_H_20_O_4_	+	313.14344	313.14249	−3.020	313.14249, 295.13171, 269.15353, **251.14236**
P_15_	TanshinoneαA isomer	55.25	C_19_H_18_O_3_	+	295.13287	295.13211	−2.578	295.13104, 267.13715, **184.01865**
P_16_	Dihydrotanshinone I	55.45	C_18_H_14_O_3_	+	279.10157	279.15836	−2.600	279.09811, 167.03360, **149.02304**
P_17_	Tanshinone I ^a^ (A_35_)	57.26	C_18_H_12_O_3_	+	277.08592	277.08524	−2.132	277.08493**, 249.09039**, 221.09573, 178.07707
P_18_	Cryptotanshinone ^a^ (A_36_)	57.32	C_19_H_20_O_3_	+	297.14852	297.14774	−2.628	279.13742,251.14249
P_19_	TanshinoneαA ^a^(A_38_)	59.12	C_19_H_18_O_3_	+	295.13287	295.13245	−1.426	295.13208, 277.12158, 249.12682,20708025
L_1_	Senkyunolide J/N isomer	22.69	C_12_H_18_O_4_	+	227.12778	227.12724	−2.402	**209.11659**, 191.10608
L_2_	Senkyunolide I/H isomer	25.86	C_12_H_16_O_4_	+	225.11213	225.11154	−2.645	**207.10107**, 165.09067, 137.09589
L_3_	Senkyunolide I/H isomer	27.68	C_12_H_16_O_4_	+	225.11213	225.11166	−2.112	**207.10107**, 165.09065, 137.09589
L_4_	Senkyunolide J/N isomer	30.45	C_12_H_18_O_4_	+	227.12778	227.12712	−2.930	249.10902, 209.11671, 191.10614, 163.11134, **153.05424**
L_5_	Senkyunolide J/N isomer	31.30	C_12_H_18_O_4_	+	227.12778	227.12721	−2.534	249.10927, 209.11664, 191.10611, 163.11128, **153.05421**
L_6_	Perloyrine	32.40	C_16_H_12_N_2_O_2_	+	265.09715	265.09641	−2.807	247.08580, 219.09067, **206.08324**, 185.07040
L_7_	Senkyunolide F isomer	32.90	C_12_H_14_O_3_	+	207.10157	207.10114	−2.080	**189.09055**, 161.09563
L_8_	Senkyunolide I/H isomer	32.92	C_12_H_16_O_4_	+	225.11213	225.11145	−3.045	247.09338, **207.10104**, 189.09039, 165.09076
L_9_	Senkyunolide J/N isomer	33.13	C_12_H_18_O_4_	+	227.12778	227.12715	−2.798	209.11665, 191.10616, 163.11128, **153.05423**
L_10_	Senkyunolide I/H ^a^ (A_21_)	34.94	C_12_H_16_O_4_	+	225.11213	225.11130	−3.712	247.09317, **207.10097**, 189.09077, 165.09033
L_11_	Senkyunolide F isomer	34.97	C_12_H_14_O_3_	+	207.10157	207.10089	−3.228	207.10103, **189.09053**, 161.09546
L_12_	Senkyunolide F isomer	36.89	C_12_H_14_O_3_	+	207.10157	207.10118	−1.887	207.10103, **189.09055**, 161.09578
L_13_	SenkyunolideB/C/E isomer	41.50	C_12_H_12_O_3_	+	205.08592	205.08531	−2.978	187.07489, 177.09053, 163.03853, **149.02296**
L_14_	E/Z-Butylidenephthalide	44.13	C_12_H_12_O_2_	+	189.09101	189.09052	−2.571	171.08003, **161.09569**, 153.06956
L_15_	SenkyunolideB/C/E isomer	44.27	C_12_H_12_O_3_	+	205.08592	205.08542	−2.442	187.07477, 163.03853, **149.02298**
L_16_	E/Z-Butylidenephthalide	50.04	C_12_H_12_O_2_	+	189.09101	189.09041	−3.153	171.07994, 153.06944, **133.02815**
L_17_	Butylidenephthalide isomer	50.04	C_12_H_12_O_2_	+	189.09101	189.09041	−0.616	189.09045,171.07994,161.09569,143.08519,**133.02815**
L_18_	SenkyunolideG/K	50.31	C_12_H_16_O_3_	+	209.11722	209.11652	−3.352	173.09526,163.11130,149.05936,145.10080,135.04381
L_19_	Ligustilides isomer	50.31	C_12_H_14_O_2_	+	191.10666	191.10603	−0.636	191.10611, 149.05936, **135.04381**
L_20_	Neocnidilide	50.59	C_12_H_18_O_2_	+	195.13796	195.13741	−2.800	177.12680, 167.14268, 159.11650, **81.07021**
L_21_	Senkyunolide A isomer	50.77	C_12_H_16_O_2_	+	193.12231	193.12196	−1.793	193.12181, 175.11171, 165.12669, 149.02275, 137.05954,**85.06510**, 57.07050
L_22_	Senkyunolide A isomer	51.72	C_12_H_16_O_2_	+	193.12231	193.12199	−1.638	165.12685, 147.11612, 137.05928, **85.06510**, 57.07080
L_23_	SenkyunolideB/C/E isomer	52.37	C_12_H_12_O_3_	+	205.08592	205.08553	−1.905	**187.07495**, 169.06419, 159.08002, 149.02292,
L_24_	Senkyunolide M isomer	52.57	C_16_H_22_O_4_	+	279.15908	279.15839	−2.492	301.14026;261.14850, **233.15289**, 215.14252, 191.10616, 173.09566, 71.049963
L_25_	Senkyunolide M isomer	53.36	C_16_H_22_O_4_	+	279.15908	279.15842	−2.385	301.14023,261.14780,251.16348,243.13681,233.15300,**191.10619**, 149.02301, 71.04964
L_26_	Senkyunolide A	53.53	C_12_H_16_O_2_	+	193.12231	193.12212	−0.965	175.11130,147.11649, **137.05946**,93.07011
L_27_	Senkyunolide M isomer	53.69	C_16_H_22_O_4_	+	279.15908	279.15836	−2.600	261.14764,**233.15303**,149.02307,105.03358,71.04965
L_28_	Senkyunolide A isomer	53.72	C_12_H_16_O_2_	+	193.12231	193.12192	−2.000	175.11131, 147.11650, **137.05945**
L_29_	Ligustilide ^a^ (A_33_)	55.41	C_12_H_14_O_2_	+	191.10666	191.10637	−1.498	**173.09579**, 163.11143, 145.10085
L_30_	Butylidenephthalide ^a^ (A_34_)	55.51	C_12_H_12_O_2_	+	189.09101	189.09077	−1.249	171.08009,161.09583,153.06960, **149.02299**, 133.02811
L_31_	Cnidumlactone B	55.74	C_24_H_30_O_5_	+	399.21660	399.21567	−2.331	421.19784, 307.16711, **191.10616**
L_32_	Levistolide A isomer	55.76	C_24_H_28_O_4_	+	381.20603	381.20499	−2.744	335.15710, 307.16754, 251.10570, **191.0612**
L_33_	Levistolide A ^a^ (A_37_)	58.88	C_24_H_28_O_4_	+	381.20603	381.20502	−2.665	381.20532, 363.19830, **191.10625**
F_1_	Catechin ^a^ (A_9_)	16.02	C_15_H_14_O_6_	−	289.07176	289.07108	−0.415	289.07120,**245.0813**0,221.08067,203.07079,179.03343,165.01825,151.03885,137.02296
F_2_	Quercetin-O-2glu/gal isomer	19.79	C_27_H_30_O_17_	+	627.15557	627.15369	−3.007	465.10092, 355.40744,**303.04895**, 127.03870, 85.02866, 69.03396
F_3_	Quercetin-O-2glu/gal isomer	21.69	C_27_H_30_O_17_	+	627.15557	627.15363	−3.102	465.10059, **303.04892**, 127.03899, 85.02874
F_4_	Quercetin-O-2glu/gal isomer	22.34	C_27_H_30_O_17_	+	627.15557	627.15338	−3.501	465.10056, **303.04895**, 127.03912, 85.02863
F_5_	Quercetin-O-2glu/gal isomer	26.42	C_27_H_30_O_17_	+	627.15557	627.15369	−3.007	465.10049,**303.04901**,287.05411,127.03870,85.02872
F_6_	Quercetin-O-2glu/gal isomer	26.78	C_27_H_30_O_17_	+	627.15557	627.15405	−2.433	627.15643,465.10114,**303.04889**,288.05804,177.05420,127.03889, 145.02821, 85.02873
F_7_	Rutin isomer	27.05	C_27_H_30_O_16_	+	611.16066	611.15894	−2.816	449.10614**, 287.05414**
F_8_	Kaempferol-O-Glc-isomer	28.86	C_21_H_20_O_11_	+	449.10784	449.10632	−3.380	**287.05411**;153.01807, 121.02850,85.02871
F_9_	Rutin isomer	28.86	C_27_H_30_O_16_	+	611.16066	611.15863	−3.323	**287.05405**, 145.04912, 85.02869
F_10_	Quercetin-isomer	29.66	C_15_H_10_O_7_	+	303.04993	303.04898	−3.132	**285.03848**, 275.01776, 257.01776
F_11_	Quercetin-isomer	30.31	C_15_H_10_O_7_	+	303.04993	303.04901	−3.033	303.01257,257.04388, **229.04872**,165.01776,153.01802
F_12_	Rutin ^a^ (A_17_)	30.31	C_27_H_30_O_16_	+	611.16066	611.15912	−2.522	**303.04904**, 153.01854
F_13_	Hyperin ^a^ (A_18_)	30.35	C_21_H_20_O_12_	+	465.10275	465.10132	−1.432	465.10117, **303.04898**, 153.12683, 135.11655, 85.02870
F_14_	Quercetin ^a^ (A_19_)	30.98	C_15_H_10_O_7_	+	303.04993	303.04932	−2.010	303.04901, 257.04413, **207.10074**, 165.01768
F_15_	Hyperin-isomer	30.99	C_21_H_20_O_12_	+	465.10275	465.10165	−1.102	465.10165, **303.04926**, 153.12700,135.11655, 85.02870
F_16_	Luteolin-glc-isomer	31.08	C_21_H_20_O_11_	+	449.10784	449.10660	−2.756	449.17566, **287.05414**
F_17_	Luteolin-O-glc ^a^ (A_20_)	31.38	C_21_H_20_O_11_	+	449.10784	449.10645	−3.090	449.17886, 391.20599, **287.05432**,
F_18_	Kaempferol-O-Glc-isomer	33.23	C_21_H_20_O_11_	+	449.10784	449.10660	−2.756	**287.05408**;153.01796, 145.04919, 127.03870,
F_19_	Kaempferol-rut	33.23	C_27_H_30_O_15_	+	595.16575	595.16406	−2.834	**287.05405**, 129.05443, 85.02871
F_20_	Kaempferol-O-Glc-isomer	34.02	C_21_H_20_O_11_	+	449.10784	449.10641	−3.179	**287.05402**, 153.01796, 121.02817
F_21_	Kaempferol-O-glu/gal + glu A	34.03	C_28_H_32_O_16_	+	625.17631	625.17426	−3.281	479.11679,**317.06464**
F_22_	Kaempferol-O-Glc-isomer	34.27	C_21_H_20_O_11_	+	449.10784	449.10687	−2.155	**287.05420**, 127.03868, 145.04906
F_23_	Luteolin/kaempferol-isomer	37.69	C_15_H_10_O_6_	+	287.05501	287.05417	−2.942	269.04379, 247.09439, 165.01772, **121.02880**,
F_24_	Quercetin-isomer	41.79	C_15_H_10_O_7_	+	303.04993	303.04898	−3.132	303.04901,257.04413,153.01775, 165.01776, **105.03358**
F_25_	Luteolin ^a^ (A_25_)	41.91	C_15_H_10_O_6_	+	287.05501	287.05408	−3.256	**153.01747**, 137.09558, 135.04381
F_26_	Naringenin ^a^ (A_27_)	45.76	C_15_H_12_O_5_	+	273.07575	273.07495	−2.930	273.07489, **153.01787**, 147.04372
F_27_	Apigenin ^a^ (A_29_)	46.95	C_15_H_10_O_5_	+	271.06010	271.05927	−3.062	271.05923, **153.01784**, 119.04916
F_28_	Kaempferol ^a^ (A_31_)	47.84	C_15_H_10_O_6_	+	287.05501	287.05411	−3.151	258.05060, 153.01787,133.02806, **121.02821**
O_1_	Succinic acid	2.29	C_4_H_6_O_4_	−	117.01933	117.018179	−9.832	117.01817, **99.00740**,73.02811
O_2_	Gallic acid ^a^ (A_1_)	2.97	C_7_H_6_O_5_	−	169.01425	169.01289	−8.026	169.01306, **125.02301**
O_3_	5-Hydroxymethylfurfural ^a^ (A_2_)	2.98	C_6_H_6_O_3_	−	125.02442	125.02327	−9.198	125.02305, 97.02824,69.03322
O_4_	1¢-*O*-galloylsucrose	3.32	C_19_H_26_O_15_	−	493.11989	493.11902	−1.771	**313.05569**, 169.01317
O_5_	Matrine ^a^ (A_3_)	3.37	C_15_H_24_ON_2_	+	249.19614	249.19579	−1.404	249.19540, **232.15358**
O_6_	6¢-*O*-galloylsucrose isomer	3.58	C_19_H_26_O_15_	−	493.11989	493.11880	−2.217	**313.05627**, 169.01279
O_7_	6¢-*O*-galloylsucrose isomer	3.78	C_19_H_26_O_15_	−	493.11989	493.11856	−2.704	**313.05624**, 169.01260
O_8_	Salvianic acid A sodium ^a^ (A_4_)	4.59	C_9_H_9_O_5_Na	+	221.04214	221.04155	−0.495	221.07767, 205.15816, 175.14740, **111.08044**
O_9_	Safflochalconeside isomer	4.67	C_21_H_20_O_10_	+	433.11292	433.11172	−2.778	415.10101, **235.02301**
O_10_	Vanillic acid isomer	4.89	C_8_H_8_O_4_	−	167.03498	167.03358	−8.394	167.03377, 149.02299, 139.03867, **123.04369**
O_11_	Safflochalconeside isomer	4.97	C_21_H_20_O_10_	+	433.11292	433.11194	−2.270	433.11240, 415.10089, 385.09070, 367.08029, 355.08008, **235.02301**
O_12_	Tanshinol ^a^ (A_5_)	4.98	C_9_H_10_O_5_	−	197.04555	197.04436	−6.022	197.04468,179.03392,**135.04376**, 123.04372
O_13_	Ethyl gallate isomer	4.98	C_9_H_10_O_5_	−	197.04555	197.04436	−6.022	197.04468, 179.03392, **135.04376**,123.04371
O_14_	Protocatechuic acid ^a^ (A_6_)	5.56	C_7_H_6_O_4_	+	155.03389	155.03360	−1.840	155.0348, **137.02309**, 111.04404
O_15_	Ethyl gallate isomer	7.74	C_10_H_12_O_5_	−	211.06120	211.0007	−5.338	211.06024, 196.03683, 181.04944, 163.03877, **151.03871**, 148.01527, 136.01520
O_16_	p-Anisicacid(4-MethoxybenzoicAcid)-isomer	7.97	C_8_H_8_O_3_	−	151.04007	151.03857	−8.590	**151.03877**, 133.02815, 123.04373, 107.04879
O_17_	Chlorogenic acid isomer	8.28	C_16_H_18_O_9_	−	353.08781	353.08694	−2.451	353.08737, **191.05504**, 179.03381, 135.04372
O_18_	Methyl gallate	8.28	C_8_H_8_O_5_	−	183.02990	183.02856	−7.303	183.02872, 168.00516, **163.03880**,135.04372
O_19_	Protocatechuic aldehyde isomer	9.54	C_7_H_6_O_3_	−	137.02442	137.02318	−9.049	137.02306, **93.03311**
O_20_	Protocatechuic aldehyde isomer	9.54	C_7_H_6_O_3_	−	137.02442	137.02324	−8.612	**137.02306**, 109.02814,93.03306
O_21_	Protocatechuic aldehyde isomer	10.52	C_7_H_6_O_3_	−	137.02442	137.02323	−8.685	137.02301, **93.03305**
O_22_	Tetramethylpyrazine ^a^ (A_8_)	11.29	C_8_H_12_N_2_	+	137.10733	137.10713	−1.422	137.10707, 122.08335
O_23_	Vanillic acid isomer	15.46	C_8_H_8_O_4_	−	167.03498	167.03360	−8.274	167.03378, **123.04375**
O_24_	Benzoic Acid	15.86	C_7_H_6_O_2_	−	121.02950	121.02841	−9.006	**121.02808**, 108.02028,94.02835
O_25_	Chlorogenic acid ^a^ (A_10_)	17.12	C_16_H_18_O_9_	−	353.08781	353.08688	−2.621	353.08743, **191.05505**, 179.03372, 135.04366
O_26_	Caffeic acid ^a^ (A_11_)	17.49	C_9_H_8_O_4_	−	179.03498	179.03365	−7.440	179.03389, 135.04378
O_27_	Safflor yellow A	18.50	C_27_H_30_O_15_	+	595.16575	595.16364	−3.539	577.15753,433.11160,147.04370
O_28_	Safflochalconeside isomer	18.50	C_21_H_20_O_10_	+	433.11292	433.11124	−3.886	415.10135, **235.02301**
O_29_	Carthamidin/isocarthamidin-glu/gal isomer	18.50	C_21_H_22_O_11_	+	451.12349	451.12189	−3.542	289.06970, 271.05914, **211.02304**
O_30_	Carthamidin/isocarthamidin-2glu/gal	18.50	C_27_H_32_O_16_	+	613.17631	613.17456	−2.856	451.12244,331.08035,289.06982,**211.02307**
O_31_	Hydroxysafflor yellow A ^a^(A_13_)	18.76	C_27_H_32_O_16_	−	611.16176	611.15911	−2.418	611.16290,491.11926,473.10776,403.10294,**325.07040**, 295.06198
O_32_	Chlorogenic acid isomer	18.92	C_16_H_18_O_9_	−	353.08781	353.08670	−3.130	353.08759, **191.05508**, 179.03381
O_33_	4-Hydroxytoluene; (4-Methylphenol)	19.70	C_7_H_8_O	−	107.05024	107.04921	−9.622	107.04878
O_34_	Tanshinol isomer	23.84	C_9_H_10_O_5_	−	197.04555	197.04437	−5.972	197.04466, **169.01309**, 125.02301
O_35_	Ethyl gallate isomer	23.84	C_9_H_10_O_5_	−	197.04555	197.04443	−5.667	197.04466, **169.01309**,125.022297
O_36_	Safflochalconeside isomer	25.48	C_21_H_20_O_10_	+	433.11292	433.11157	−3.124	415.10184,397.09003,367.08011, **277.03345**
O_37_	Coniferyl aldehyde, (ferulaldehyde)	26.03	C_10_H_10_O_3_	−	177.05572	177.05432	−7.949	177.05499, 162.03194, 149.05939, **129.01839**, 105.03781, 99.00752,71.01251
O_38_	Ferulic Acid ^a^ (A_16_)	27.09	C_10_H_10_O_4_	+	195.06519	195.06482	−1.873	195.06458, **177.0541**8, 145.02809, **135.04388**
O_39_	Vanillin	28.63	C_8_H_8_O_3_	−	151.04007	151.03857	−9.914	**151.03877**, 135.00745, 109.02804
O_40_	p-Anisic acid (4-Methoxybenzoic Acid)	28.63	C_8_H_8_O_3_	−	151.04007	151.03856	−9.980	**151.03877**, 135.00745, 109.02804
O_41_	Protocatechuic aldehyde ^a^ (A_7_)	28.68	C_7_H_6_O_3_	−	137.02442	137.02314	−9.341	137.02304, **93.03307**
O_42_	Carthamidin/isocarthamidin-glu/gal isomer	28.76	C_21_H_22_O_11_	+	451.12349	451.12207	−3.143	**289.06793**, 169.01270, 147.04370
O_43_	Tetragalloyl glucose	28.81	C_34_H_28_O_22_	−	787.09994	787.09705	−3.679	787.09833,465.06750, 295.04462, **169.01303**
O_44_	Carthamidin/isocarthamidin-glu/gal isomer	31.70	C_21_H_22_O_11_	+	451.12349	451.12238	−2.456	**289.06793**, 169.01265,147.04366, 85.02869
O_45_	Pentagalloylglucose	32.83	C_41_H_32_O_26_	−	939.11989	939.10724	−3.902	939.11371,769.08820, 617.08051, 447.05655, 295.04709, **169.01294**
O_46_	Lithospermic acid isomer	33.03	C_27_H_22_O_12_	−	537.10385	537.10175	−3.908	537.10107,519.09387,375.06934,339.05014,295.06058,201.01610,179.03391,**161.02318**
O_47_	Lithospermic acid isomer	33.75	C_27_H_22_O_12_	−	537.10385	537.10022	−6.757	537.09991,**375.06915**,357.05890,201.01595, 179.03377
O_48_	Azelaic acid	33.95	C_9_H_16_O_4_	−	187.09758	187.09633	−6.693	187.09651, 169.08598, 143.10638, **125.09573**
O_49_	Carthamidin/isocarthamidin-glu/gal isomer	35.33	C_21_H_22_O_11_	+	451.12349	451.12234	−2.545	304.09924, **289.06793**, 169.01265
O_50_	Lithospermic acid isomer	35.77	C_27_H_22_O_12_	−	537.10385	537.10040	−6.422	**375.06982**, 201.01608, 179.03377, 135.04445
O_51_	Rosmarinic acid ^a^ (A_22_)	35.88	C_18_H_16_O_8_	−	359.07724	359.07632	−2.564	359.07629, 197.04462, 179.03386, **161.02316**
O_52_	Salvianolic acid A isomer	37.03	C_26_H_22_O_10_	−	493.11347	493.11267	−2.738	**295.06073**,197.04436,179.03427,109.02807
O_53_	Lithospermic acid isomer	37.03	C_27_H_22_O_12_	−	537.10385	537.10297	−1.637	**295.06070**, 179.03328, 109.02803
O_54_	Crocin I ^a^ (A_23_)	38.77	C_44_H_64_O_24_	−	975.37148	975.37061	−1.849	**651.26538**, 327.16083, 283.17017
O_55_	Salvianolic acid B ^a^ (A_24_)	39.18	C_36_H_30_O_16_	−	717.14611	717.14417	−2.702	519.09308,339.05045,**321.04007**,295.06061, 249.05511
O_56_	3,7- or 3,8-Dimethyl ellagic acid isomer	42.65	C_16_H_10_O_8_	−	329.03029	329.02939	−2.737	329.03018, **314.00659**,298.98291
O_57_	Salvianolic acid A ^a^ (A_26_)	43.42	C_26_H_22_O_10_	−	493.11347	493.11261	−2.859	**295.06076**,185.02339, 109.02803
O_58_	3,7- or 3,8-Dimethyl ellagic acid isomer	43.57	C_16_H_10_O_8_	−	329.03029	329.02945	−2.554	329.02982, **314.00644**, 298.98282, 270.98758
O_59_	Ethyl4-hydroxy-3-methoxycinnamate	44.31	C_12_H_14_O_4_	−	221.08193	221.08087	−4.805	221.08109, **177.09096**
O_60_	Salvianolic acid C isomer	44.92	C_26_H_20_O_10_	−	491.09837	491.09723	−2.321	311.05563, **293.04517**
O_61_	Paeonol^a^(A_28_)	46.25	C9H10O3	+	167.07027	167.07008	−1.142	167.06992,149.05942,121.06463,109.02848
O_62_	Salvianolic acid C isomer	48.31	C_26_H_20_O_10_	−	491.09837	491.09741	−1.955	311.05563, **293.04517**
O_63_	Ethyl ferulate^a^(A_32_)	50.72	C_12_H_14_O_4_	−	221.08193	221.08078	−5.212	221.08099,177.09093,149.09587,134.03592121.02803, **71.04876**,69.03313

a:Structures confirmed by comparison with reference standards, and A1-A38 were the mark number of reference standards.

**Bold characters**: the base peaks in MS^n^ spectra.

### Identification of monoterpene glycosides in XBJ

19 monoterpene glycosides were identified and listed in Table 1. M_1_ gave [M−H]^−^ ion at *m/z* 359.13400 (C_16_H_23_O_9_) in full scan mass spectrum. In it’s MS/MS^2^ experiment, the obtained ion produced characteristic fragment of [M−H−Glc]^−^ at *m/z* 197.08099 (C_10_H_13_O_4_), corresponding to the paeonisuffrone, was observed, the further loss of H_2_O group generated the fragment of [M−H−Glc−H_2_O]^−^ at *m/z* 179.07028 (C_10_H_11_O_3_) was also observed. Thus, M_1_ was deduced as 1-*O*-*β*- d-glucopyranosyl-paeoni-suffrone. Three isomers (M_4_, M_5_ and M_7_) revealed the same [M−H]^−^ ions at *m/z* 527.13922 (C_23_H_27_O_14_). In the MS/MS^2^ experiment of M_4_, the [M−CH_2_OH]^−^ ion at *m/z* 497.12943 (C_22_H_25_O_13_) and [M−CH_2_OH−H_2_O]^−^ ion at *m/z* 479.11896 (C_22_H_23_O_12_) was produced by the loss of CH_2_OH unit, and the further loss of H_2_O. The precursor ion of M_4_ generated fragment at *m/z* 313.05627 (C_13_H_13_O_9_) by loss of the aglycone moiety, and the further loss of hexose moiety produced the galloyl fragment at *m/z* 169.01294 (C_7_H_5_O_5_). M_5_ had the same ions at *m/z* 313.05627 and 169.01294 in its MS/MS spectrum with M_4_ and M_7_. M_4_, M_5_ and M_7_ were identified as 6′-*O*-galloyl Desbenzoylpaeoniflorin and its isomers.

Four isomers (M_12_, M_14_, M_15_ and M_16_) revealed the same [M−H]^−^ ions at *m/z* 631.16498 (C_30_H_31_O_15_). In the MS/MS spectrum of M_12_, the loss of H_2_O group from precursor ion at *m/z* 613.15570 (C_30_H_29_O_14_) and the further loss of benzoyl group at *m/z* 491.11874 (C_23_H_23_O_12_) was observed. The obtained ion produced fragment corresponding to galloyl attached at one hexose moiety at *m/z* 313.05603 (C_13_H_13_O_9_), and the galloyl fragment at *m/z* 169.01294 (C_7_H_5_O_5_) were found. All of M_14_, M_15_, and M_16_ had the same fragments at *m/z* 313.05603 (C_13_H_13_O_9_), at *m/z* 169.01294 (C_7_H_5_O_5_), and benzoyl group at *m/z* 121.02809 (C_7_H_5_O_2_) in their respective MS/MS spectra. M_12_, M_14_, M_15_, and M_16_ were identified as galloylpaeoniflorin and its isomers. M_17_ and M_18_ showed the same [M−H]^−^ ion at *m/z* 599.17554 (C_30_H_31_O_13_). Apart from the characteristic fragments of paeoniflorin, both of their MS/MS spectra displayed the fragment at *m/z* 477.13870 (C_23_H_25_O_11_), 281.06613 (C_13_H_13_O_7_), 137.02303 (C_7_H_5_O_3_), 121.02802 (C_7_H_5_O_2_), indicating the existence of O-benzoyl unit, benzoyl unit, and hexose moiety. M_17_ and M_18_ were deduced as benzoyloxypaeoniflorin and its isomer. M_19_ displayed the [M−H]^−^ ion at *m/z* 583.18210 (C_30_H_31_O_12_), which had one less oxygen than that of M_17_ and M_18_. By comparing and the analysis of their MS/MS spectra, the absence of ion at *m/z* 137.02303 (C_7_H_5_O_3_), and the presence of ion at *m/z* 121.02801 (C_7_H_5_O_2_), indicated the benzoyl unit in M_19_ instead of O-benzoyl unit in M_17_ and M_18_. Thus, M_19_ was identified as benzoylpaeoniflorin.

### Identification of phenanthrenequinone in XBJ

19 phenanthrenequinone were identified and listed in Table 1. P_1_ displayed a [M + H]^+^ ion at *m/z* 297.11133 (C_18_H_17_O_4_). In the MS/MS^2^ experiment, the obtained ion produced [M−H_2_O]^+^ fragment at m/z 279.15570(C_18_H_15_O_3_) and [M-2H_2_O]^+^ ion at *m/z* 261.09055 (C_18_H_13_O). P_1_ was identified as tanshinone VI. Four isomers P_3_, P_4_, P_5_, and P_14_ revealed the same [M + H]^+^ ion at *m/z* 313.14249 (C_19_H_21_O_4_). In the MS/MS^2^ experiment, the obtained ion produced [M−H_2_O]^+^ fragment at *m/z* 295.13196 (C_19_H_19_O_3_), [M−CO_2_]^+^ ion at *m/z* 269.15262 (C_18_H_21_O_2_), and [M − H_2_O−CO_2_]^+^ ion at *m/z* 251.14232 (C_18_H_19_O) were found in P_3_. P_14_ had the same fragment ions with P_3_. In the MS/MS spectrum of P_4_, [M−H_2_O]^+^, [M−2H_2_O]^+^, and [M−H_2_O−CO_2_]^+^ ions at *m/z* 295.13177 (C_19_H_19_O_3_), *m/z* 277.12137 (C_19_H_17_O_2_), *m/z* 251.14224 (C_18_H_19_O_3_) were detected. The fragment of [M−CO]^+^, and [M−CO−H_2_O]^+^ ions at *m/z* 285.14700 (C_18_H_21_O_3_), and *m/z* 267.13751 (C_18_H_19_O_2_) were observed in the MS/MS^2^ experiment of P_5_. This fragmentation information were similar with that of phenanthrenequinone, and their molecular was accordance with tanshinone II B, the major constituent in tanshin, one composition of traditional Chinese medicine in XBJ. Thus, P_3_, P_4_, P_5_, and P_14_ were deduced as tanshinone II B and its isomers.

P_8_ gave a [M + H]^+^ ion at *m/z* 299.16432 (C_19_H_23_O_3_). Its MS/MS experiment generated [M−H_2_O]^+^ ion at *m/z* 281.15341 (C_19_H_21_O_2_), and the further loss of CO produced the [M−H_2_O−CO]^+^ ion at *m/z* 253.15788 (C_18_H_21_O). P_8_ was identified as miltiodiol. P_9_ exhibited a [M + H]^+^ ion at *m/z* 299.16339 (C_19_H_23_O_3_) in the positive full scan mode. The fragment at *m/z* 281.15314 (C_19_H_21_O_2_) indicated the loss of H_2_O from the precursor ion. The other product ion at *m/z* 253.15788 (C_18_H_21_O) revealed the further splitting of a CO_2_ group. This information led to the conclusion that P_9_ was deoxyneocryptotanshinone. Two isomers P_13_ and P_18_ showed the same [M + H]^+^ ion at *m/z* 297.14852. The MS/MS experiment of P_13_ generated [M−CO_2_]^+^ ion at *m/z* 249.09039 (C_18_H_21_O). P_13_ were identified as Cryptotanshinone by comparing with the retention time and high-resolution accurate mass and P_18_ was identified as its isomers.

### Identification of lactones in XBJ

The detailed MS data of 33 lactones were listed in Table 1. Four isomers L_1,_ L_4,_ L_5_ and L_9_ revealed the same [M + H]^+^ ions at *m/z* 227.12724 (C_12_H_19_O_4_). In their MS/MS spectrum, the characteristic fragment ions of senkyunolide J/N, such as 209.11671 (C_12_H_17_O_3_), 191.10614 (C_12_H_15_O_2_,), 163.11134 (C_11_H_15_O), 153.05424(C_8_H_9_O) were observed. So they were assigned as senkyunolide J/N and its isomers. Similarly, L_2_, L_3_, L_8_ and L_10_ were identified as senkyunolide I/H and its isomers owing to the presence of diagnostic fragment ions related to senkyunolide I/H. L_7_, L_11_, and L_12_ showed the same [M + H]^+^ ion at *m/z* 207.10114 (C_19_H_21_O_4_) in their full scan positive mass spectrum. In their MS/MS spectra, the same ions at *m/z* 189.09053 (C_12_H_13_O_2_) produced by the loss of H_2_O group from precursor ion, and *m/z* 161.09546 (C_11_H_13_O) produced by the further loss of CO group were found. These characteristic information related to senkyunolide suggested L_7_, L_11_, and L_12_ to be senkyunolide F and its isomers. L_13_, L_15_, and L_23_ were determined as senkyunolide B/C/E, for the characteristic fragment ions of senkyunolide B/C/E, at *m/z* 187.07489 (C_12_H_11_O_2_), 177.09053 (C_11_H_13_O_2_,), and 163.03853 (C_9_H_7_O_3_), 149.02296 (C_8_H_5_O_3_). L_18_ exhibited [M + H]^+^ ion at *m/z* 209.11652 (C_12_H_17_O_3_) in its full scan positive mass spectrum, indicating the molecular formula of C_12_H_16_O_3._ The MS/MS experiment of L_18_ generated [M−2H_2_O]^+^ ion at *m/z* 173.09526 (C_12_H_13_O) by successive loss of H_2_O group from the precursor ion. L_18_ was identified as senkyunolide G/K. L_21_, L_22_, L_26_ and L_28_ exhibited the same [M + H]^+^ ion at *m/z* 193.12196 (C_12_H_17_O_2_) in positive ion mode, indicating the molecular formula of C_12_H_17_O_2_, which had one less oxygen atom than that of L_18_. They had one less hydroxyl than that of L_18_ in the structure, which was verified by the fragments at *m/z* 175.11171 (C_12_H_15_O), *m/z* 165.12669 (C_11_H_17_O), *m/z* 137.05954 (C_8_H_9_O_2_) in their MS/MS spectra. Thus, L_21_, L_22_, L_26_, and L_28_ were identified as senkyunolide A and its isomers. The same [M + H]^+^ ions at *m/z* 279.15839 (C_16_H_23_O_4_) of L_24_, L_25_, and L_27_ revealed the molecular formula of C_16_H_22_O_4_. The fragment ions at *m/z* 261.14850 (C_16_H_21_O_3_), 233.15289 (C_15_H_21_O_2_), 215.14252 (C_15_H_19_O), were generated by loss of H_2_O, further loss of CO, and further loss of H_2_O, respectively. The ion at *m/z* 191.10616 (C_12_H_15_O_2_) was produced by three times of successive loss of H_2_O from fragment of [M-H_2_O-CO]^+^ at *m/z* 233.15289 (C_15_H_21_O_2_), and the further loss of H_2_O generated ion at *m/z* 173.09566 (C_12_H_13_O). These ions are the characteristic neutral losses associated with the senkyunolide M. Thus, L_24_, L_25_ and L_27_ were indicated as senkyunolide M and its isomers.

### Identification of flavonoids in XBJ

28 flavonoids were detected and deduced in positive ion mode. The detailed fragmentation information of flavonoids was listed in Table 1. F_7_, F_12_ and F_9_ displayed the same [M + H]+ ion at m/z 611.15912 (C_27_H_31_O_16_), and they were deduced as rutin and its isomers, based on the presence of diagnostic fragment ions at m/z 303.04904 (C_15_H_11_O_7_), 153.01854 (C_7_H_5_O_4_). Five isomers of F_8_, F_16_, F_18_, F_20_ and F_22_ displayed the same [M + H]+ ion at m/z 449.10632 (C_21_H_21_O_11_) with luteolin-O-glc of F_17_. In their MS/MS spectra, by lossing of the hexose moiety generated the [M−H−Glc]^+^ ion at *m/z* 287.05411 (C_15_H_11_O_6_), corresponding to the aglycone of kaempferol or luteolin. F_8_, F_16_, F_18_, F_20_, and F_22_ were identified as hexose glycoside of kaempferol or its isomers. F_23_, F_25_ and F_28_ showed the same [M + H]^+^ ion at *m/z* 287.05420 (C_15_H_11_O_6_). In their MS/MS spectra, the characteristic fragments at *m/z* 153.01807 (C_7_H_5_O_4_), 133.02815 (C_8_H_5_O_2_), and 121.02580 (C_7_H_5_O_2_), which were produced by the two different reaction routines of RDA cleavage, were observed. The loss of CO moiety from precursor ion generated the ion *m/z* 258.05179 (C_14_H_10_O_5_) was also found. Comparing with the retention time of reference solution, F_25_ was confirmed as luteolin and F_28_ was kaempferol. The characteristic ions of kaempferol are *m/z* 258.05060, 153.01787, 133.02806 and 121.02821, and the characteristic ions of luteolin are *m/z* 153.01747, 137.09558 and 135.04381.

### Identification of phenolic acids and other compounds in XBJ

The MS data of 63 detected phenolic acid and other compounds were listed in Table 1. O_12_ and O_34_ showed the same [M−H]^−^ ion at *m/z* 197.04436 (C_9_H_9_O_5_) in the negative full scan mode. Both of their MS/MS spectra displayed the [M−H_2_O]^−^ and [M−H_2_O−COOH]^−^ ions at *m/z* 179.03392 (C_9_H_7_O_4_) and 135.04376 (C_8_H_7_O_2_) suggested that O_12_ and O_34_ were Tanshinol and its isomer. O_17_, O_25_ and O_32_ displayed the same [M−H]^−^ ion at *m/z* 353.08688 (calculated 353.08781, error, −2.621 ppm) in negative full scan mode. Their MS/MS spectra showed similar ions at *m/z* 179.03372 (C_9_H_7_O_4_) and 135.04366 (C_8_H_7_O_2_). This fragmentation was associated with that of caffeic acid. O_17_, O_25_ and O_32_ were identified as chlorogenic acid and its isomers. Four isomers of O_19_, O_20_, O_21_ and O_41_ showed the same [M + H]^+^ ion at *m/z* 137.02442(C_7_H_6_O_3_). The MS/MS experiment of O_41_ generated [M−CO_2_]^+^ ion at *m/z* 93.03307 (C_6_H_5_O). O_41_ were identified as Cryptotanshinone by comparing with reference, and the O_19_, O_20_ and O_21_ were identified as its isomers. In the negative full scan mode, O_26_ showed [M−H]^−^ ion at *m/z* 179.03365 (C_9_H_7_O_4_). The MS/MS experiment yielded [M−COOH]^−^ ion at *m/z* 135.04378 (C_8_H_7_O_2_). O_26_ was identified as caffeic acid. O_53_ showed [M−H]^−^ ion at *m/z* 359.07632 (C_18_H_15_O_8_). In the MS/MS experiment, the ion at *m/z* 179.03386 (C_9_H_7_O_4_) was triggered by the loss of caffeic acid residue. Further fragment at m/z 197.04462 (C_9_H_9_O_5_) suggested the existence of acid. Therefore, O_53_ was identified as rosmarinic acid.

### Quantitative analysis of samples

A thorough and complete method validation for assaying 38 bioactive compounds in XBJ was done referring to ICH guidelines^24^. The UHPLC-Q-Orbitrap mass spectrometry was validated with respect to linearity, sensitivity, accuracy and precision, reproducibility and stability.

## Method Validation

### Linearity, LOD and LOQ

Standard stock solutions containing 38 analytes were prepared and diluted to seven appropriate concentrations for the construction of the calibration curves. Each solution was injected in triplicate, and then the linear regression equation was obtained by plotting the analyte peak area (*Y*) vs a series of analyte concentrations (*X*). The regression equation, coefficient of determination (*R* ^2^) and linear range are given in Supplementary Table S1. All the analytes showed good linearity with *R* ^2^ more than 0.9994 in the concentration range. The LOD and LOQ under the optimized chromatographic conditions were evaluated at a signal-to-noise ratio (S/N) of 3 and 10, respectively. The values of LODs and LOQs were in the range of 0.01~35.77 ng·mL^−1^ and 0.03~119.22 ng·mL^−1^, respectively (Supplementary Table S1).

### Accuracy and precision

The precision of the established method was evaluated by intra-day and inter-day variability, and the relative standard deviations (RSD) were taken as a measure. The mixed standard solution at middle concentrations was analyzed in six replicates within one day and on 3 consecutive days. The results are shown in Supplementary Table S1, and the RSD values of the intra-day and inter-day of 38 compounds were all less than 2.97%, which showed good precision of the developed method.

The accuracy of the established method was evaluated by recovery test and RE (relative error). The samples were spiked with three concentration levels (80, 100, and 120%) of known amounts of 38 reference compounds. The spiked samples of each concentration were analyzed in triplicate. The accuracy was calculated as the quotient of the measurement and the nominal value of the analyte added to the sample. The detailed accuracy data is presented in Supplementary Table S2. The mean recoveries were ranged from 98.5% to 101.3% with RSDs less than 2.98%.

### Reproducibility and Stability

In order to confirm the reproducibility, six different samples from the same batch sample were analyzed within one day and on three consecutive days. The RSDs were used as a measure and the acceptance criterion should be within 5.0%. The results are shown in Supplementary Table S1 and the RSD values of 38 compounds were all less than 3.0%, which showed good reproducibility of the developed method.

The stability of the sample solution was analyzed at room temperature on three consecutive days. The stability of the standard solutions stored at 4 °C was also examined on three consecutive days. Injections were performed at 0, 12 hour, 1, 2, 3, 5, and 7 days. The stability RSD values of 38 compounds in the sample solution were all less than 2.86% and those in standard solutions were all less than 2.0%, which showed that all analytes in the sample solution (at room temperature) and the standard solutions (at 4 °C) were found to be very stable.

## Analysis of chemical profile of XBJ sample

The developed UHPLC-Q-Orbitrap HRMS method was adopted for the routine screening of the 38 bioactive compounds in 10 XBJ samples. 38 bioactive compounds were unambiguously identified by comparing the retention times and high-resolution accurate mass of reference standards. The polarity switching in full scan modes of UHPLC-Q-Orbitrap HRMS was used to achieve the highest response intensities of various types of constituents. In addition, the Q-Orbitrap HRMS as a powerful high resolution mass spectrometry, has the function of qualitative and quantitative simultaneously, namely compounds could be qualitative and quantitative in one analysis. Table 2 showed the obtained quantitative results of each compound calculated according to calibration curves. The results shows that two compounds (Hydroxysafflor yellow A and Paeoniflorin) are the predominant constituents obviously, the contents of which are much higher than other compounds. Hydroxysafflor yellow A and Paeoniflorin are two major marker components in Carthami Flos and Paeoniae Radix Rubra. Moreover, the Q-Orbitrap HRMS has very high sensitivity, so the low-content compounds, such as Levistolide A, Tetramethylpyrazine, Butylidenephthalide and Tanshinone I, were investigated simultaneously. Thus, the constituents with high and low levels contents could be quantified in one analysis.

**Table 2:** Quantitative analytical results for 38 compounds in XBJ from 10 batches (n = 3, μg/mL).

Compounds	1500181	1504101	1504111	1504121	1505211	1505671	1508171	1508191	1509082	1509132
A_1_	7.203	6.266	5.557	5.343	6.995	5.435	5.179	6.339	7.117	6.886
A_2_	14.537	12.644	10.790	10.827	13.447	14.746	10.530	12.477	11.308	13.658
A_3_	0.086	0.099	0.145	0.099	0.116	0.095	0.095	0.150	0.105	0.118
A_4_	0.806	0.742	0.629	0.636	0.697	0.706	0.731	0.699	0.823	0.731
A_5_	3.010	2.840	2.592	2.396	3.122	3.060	2.789	2.910	2.695	2.892
A_6_	4.471	4.354	4.214	4.355	4.196	4.023	4.130	3.929	3.632	4.536
A_7_	4.974	4.430	4.480	4.138	4.164	4.890	4.654	4.202	4.521	4.733
A_8_	0.004	0.003	0.003	0.004	0.003	0.003	0.004	0.005	0.004	0.004
A_9_	4.334	4.508	4.739	5.567	4.895	5.181	6.423	4.640	6.459	6.253
A_10_	3.398	3.618	3.521	3.107	2.778	3.202	3.904	3.601	3.701	3.254
A_11_	4.733	5.033	5.057	4.366	4.619	4.923	5.071	5.170	4.467	4.848
A_12_	36.136	38.274	39.614	37.579	38.264	38.977	37.833	37.461	38.625	38.148
A_13_	587.385	599.696	608.705	550.898	517.190	614.478	608.009	620.303	590.146	593.456
A_14_	16.582	19.526	17.490	15.570	20.358	15.286	17.695	17.465	20.055	15.971
A_15_	893.515	925.366	880.513	870.379	874.547	906.911	886.107	884.818	818.990	875.418
A_16_	30.993	29.554	28.278	25.810	29.195	26.904	29.529	30.674	31.704	29.231
A_17_	4.010	3.723	4.159	3.394	3.326	3.765	3.470	3.267	3.413	3.732
A_18_	0.404	0.420	0.503	0.419	0.497	0.487	0.458	0.404	0.446	0.366
A_19_	1.224	1.230	1.297	1.003	1.057	1.075	0.998	1.093	0.871	1.117
A_20_	1.115	1.144	1.271	1.020	0.930	0.995	0.932	0.904	1.176	1.037
A_21_	85.282	84.158	84.915	84.707	85.356	93.482	90.544	79.375	83.856	83.856
A_22_	5.960	5.392	5.522	4.644	5.406	5.376	6.157	6.139	6.115	5.783
A_23_	1.654	1.882	1.800	1.517	1.520	1.546	1.518	1.620	1.669	1.669
A_24_	2.261	2.090	1.940	1.595	2.017	2.164	2.344	2.448	2.377	2.067
A_25_	0.124	0.105	0.126	0.123	0.106	0.119	0.109	0.131	0.124	0.117
A_26_	0.039	0.047	0.047	0.037	0.049	0.041	0.059	0.039	0.037	0.040
A_27_	0.350	0.495	0.476	0.418	0.360	0.328	0.412	0.376	0.386	0.330
A_28_	0.025	0.029	0.020	0.025	0.022	0.025	0.024	0.023	0.020	0.027
A_29_	0.451	0.458	0.459	0.393	0.424	0.433	0.421	0.433	0.403	0.403
A_30_	30.026	35.814	33.435	30.979	35.207	31.965	30.470	29.264	29.210	31.023
A_31_	0.206	0.213	0.219	0.231	0.242	0.221	0.200	0.236	0.282	0.205
A_32_	0.369	0.368	0.398	0.383	0.387	0.379	0.381	0.382	0.477	0.375
A_33_	0.196	0.246	0.250	0.194	0.183	0.209	0.176	0.187	0.179	0.148
A_34_	0.019	0.015	0.017	0.017	0.020	0.016	0.017	0.020	0.015	0.014
A_35_	0.020	0.021	0.021	0.016	0.022	0.020	0.022	0.018	0.023	0.023
A_36_	0.473	0.561	0.566	0.515	0.586	0.525	0.588	0.543	0.555	0.524
A_37_	0.00046	0.00047	0.00049	0.00047	0.00058	0.00055	0.00057	0.00046	0.00054	0.00050
A_38_	0.084	0.078	0.080	0.080	0.078	0.088	0.069	0.085	0.072	0.085

The RSD of total amounts of investigated 38 compounds in 10 batches XBJ samples was 2.81%, which showed good stability of the total content. However, significant variations were observed as well. The RSD of each compound in 10 batches XBJ samples was in range of 2.48% to 19.43%, which showed instability of the some compounds. However, multiple active components, including macro- and micro-components, are frequently considered to be responsible for the therapeutic effects^25^. So, the present analysis of multiple components is more reasonable for quality control of XBJ injection.

## Quality assessment of XBJ with the established strategy

### Fingerprinting

Fingerprinting strategies are internationally accepted as an acceptable means of quality control (QC) for TCMs^26^. There are significant advantages of using fingerprinting strategies for sample differentiation, as fingerprinting not only determines the characteristic patterns of each plant type but also reveals the inherent relationships between multiple compounds. The good precision, reproducibility, stability of UHPLC-Q-Orbitrap HRMS analysis were demonstrated. The chromatograms Xcalibur raw files of ten batches sample was imported into the SIEVE software. The batch of 1500181 was selected as reference chromatogram. In order to focus on the most effective information, time windows of 0–60 min was selected to generate chromatographic fingerprinting. The similarity values obtained by SIEVE software was calculated through the overall evaluation of 10 batches total ion current chromatograms. The identical peaks in 10 batches sample chromatograms can be matched in automatic and proceeded peak alignment. The retention time and peak area of all peak in 10 batches sample make a comparison with the reference chromatogram. The correlation coefficients of all introduced chromatograms relative to that of reference chromatogram would be calculated. The similarity values of 10 samples (No.1500181, 1504101, 1504111, 1504121, 1505211, 1505671, 1508171, 1508191, 1509082 and 1509132) in fingerprintings in positive and negative mode were 1, 0.990, 0.988, 0.990, 0.991, 0.989, 0.991, 0.988, 0.977 and 0.993, respectively. The similarity values were all more than 0.9 in positive and negative mode, which indicated that the samples from different batches had strong similarities with high correlation coefficients of similarities. To some degree, this results demonstrate that the fingerprinting chromatograms of these samples might be used to assess the quality of XBJ injection.

### Principal component analysis

**Figure 4:**
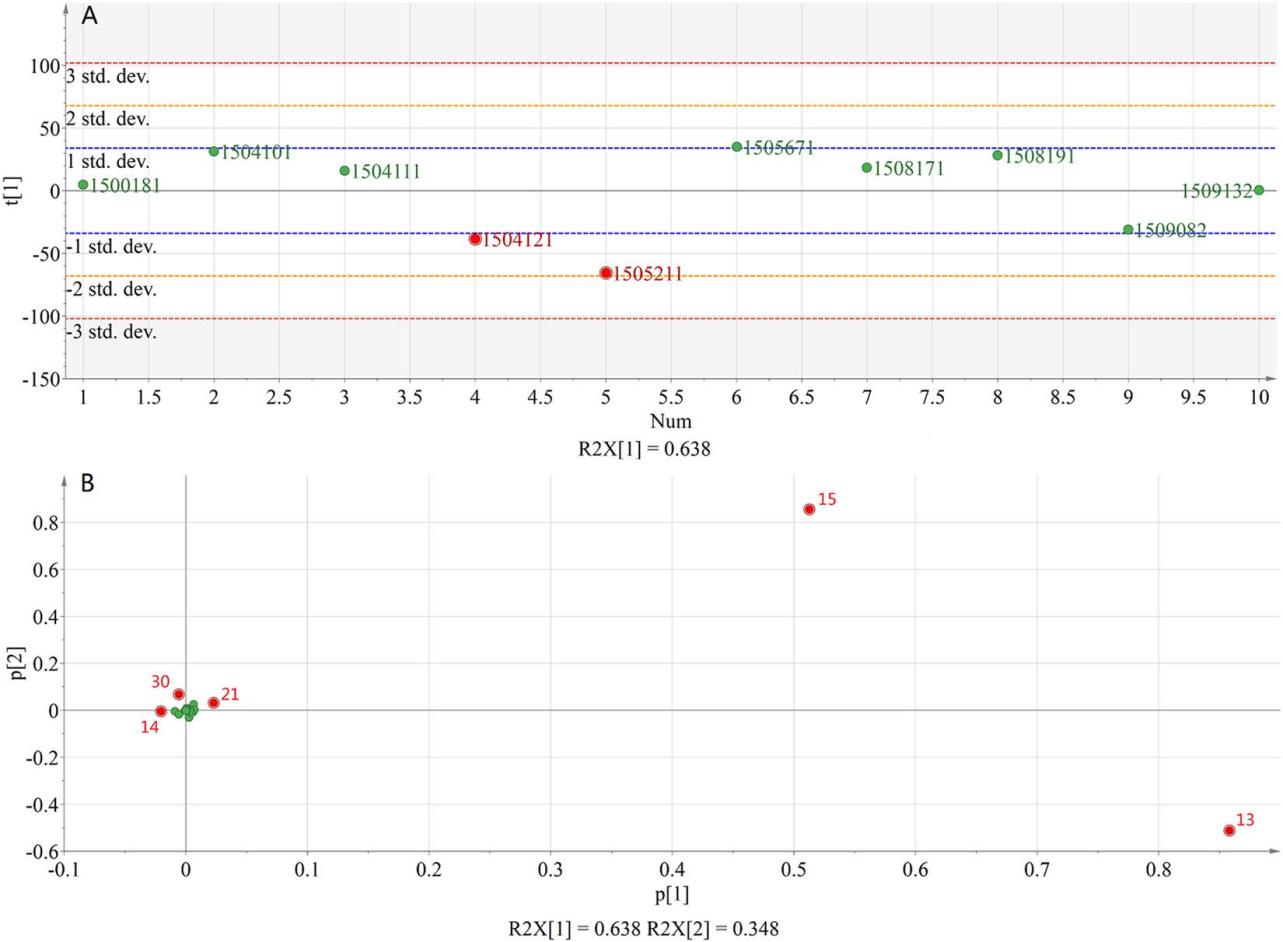
(**A**) PCA Score Scatter Plot. (**B**) PCA Loading Column Plot.

PCA was used to further classify the 10 samples. PCA is an analytical method that is used to reduce a large set of variable into a smaller set of “artificial”variables known as principal components (PCs), which account f or most of the variance in the original variables. In the present analysis, the data matrix of ten batches samples and 38 bioactive compounds was imported into the multivariate statistical analysis software SIMCA 14.0. The PCA-X model was adopted to match the data and the original 38 variable dimension generated 2 new variables through software automatically, that is the two principal components. After the data fitting, the principal component 1 of variable was accounted for larger percentage of 63.8%, which could reflect the main characteristics of the original data. So PC1 would be suitable for revealing correlations among the different variables. The Score Scatter Plot (Fig. 4A) is used to evaluate the stability of 10 batches XBJ samples. The deviation represented the degree of stability. The deviation represented the degree of stability. The smaller the deviation in the PC_1_ axis, the better stability. The Fig. 4A shows the bias of 10 batches was within ± 2 SD, indicating the quality of 10 batches was more stable. In addition, the bias of 8 batches in 10 batches was within ± 1 SD, while the bias of 2 batches in 10 batches was ranged from ± 1 SD to ± 2 SD.

The PCA Loading Plot could reflect the weight size of original variable in the principal component analysis. The greater the absolute value of original variable in the PCA Loading Plot, the more importance role of original variable in the overall distribution. So the PCA Loading Plot can make it possible to discover the variables leading to the difference. In the Loading Column Plot (Fig. 4B) of the scores, the variables of 13 and 15 were the farthest from the origin on the PC_1_ and PC_2_. A13 and A15 as the important quality markers, has a relationship with different batch of drugs on the scatter plot distribution location. In the Fig. 4B, A13 and A15 were positive and the absolute value is larger in Loading Plot on PC1, which make the most batch in the positive quadrant portion of the Score Scatter Plot and keep positively correlated with them. Because the level of A13 and A15 is lower than the average level, the batch 1505211 has obvious anomaly in the overall distribution. So the two components made this batch negatively correlated and this batch was spotted in the negative quadrant part of the Score Scatter Plot. That is to say that two markers responsible for the cluster formation were mainly compounds (A_13_ and A_15_) that suggested that the contents of Hydroxysafflor yellow A and Paeoniflorin had a significant relationship with quality of XBJ injection. In addition, the variables of 14, 21 and 30 had a certain statistical significance compared with other variables. The compounds were Albiflorin, Senkyunolide I/H and Benzoylpaeoniflorin, respectively. This three compounds could provide some reference meaning for quality evaluation of XBJ injection. In 2013 edition Drug Standards of China, Hydroxysafflor yellow A and Paeoniflorin were selected as markers due to the two highest levels chemical composition. However, the therapeutic effects are frequently considered to be connected with multiple active components, including macro- and micro-components. So, the five markers, Hydroxysafflor yellow A, Paeoniflorin, Albiflorin, Senkyunolide I/H and Benzoylpaeoniflorin, were more meaningful for the quality of XBJ injection.

### Assay of the five markers in XBJ sample

**Figure 5:**
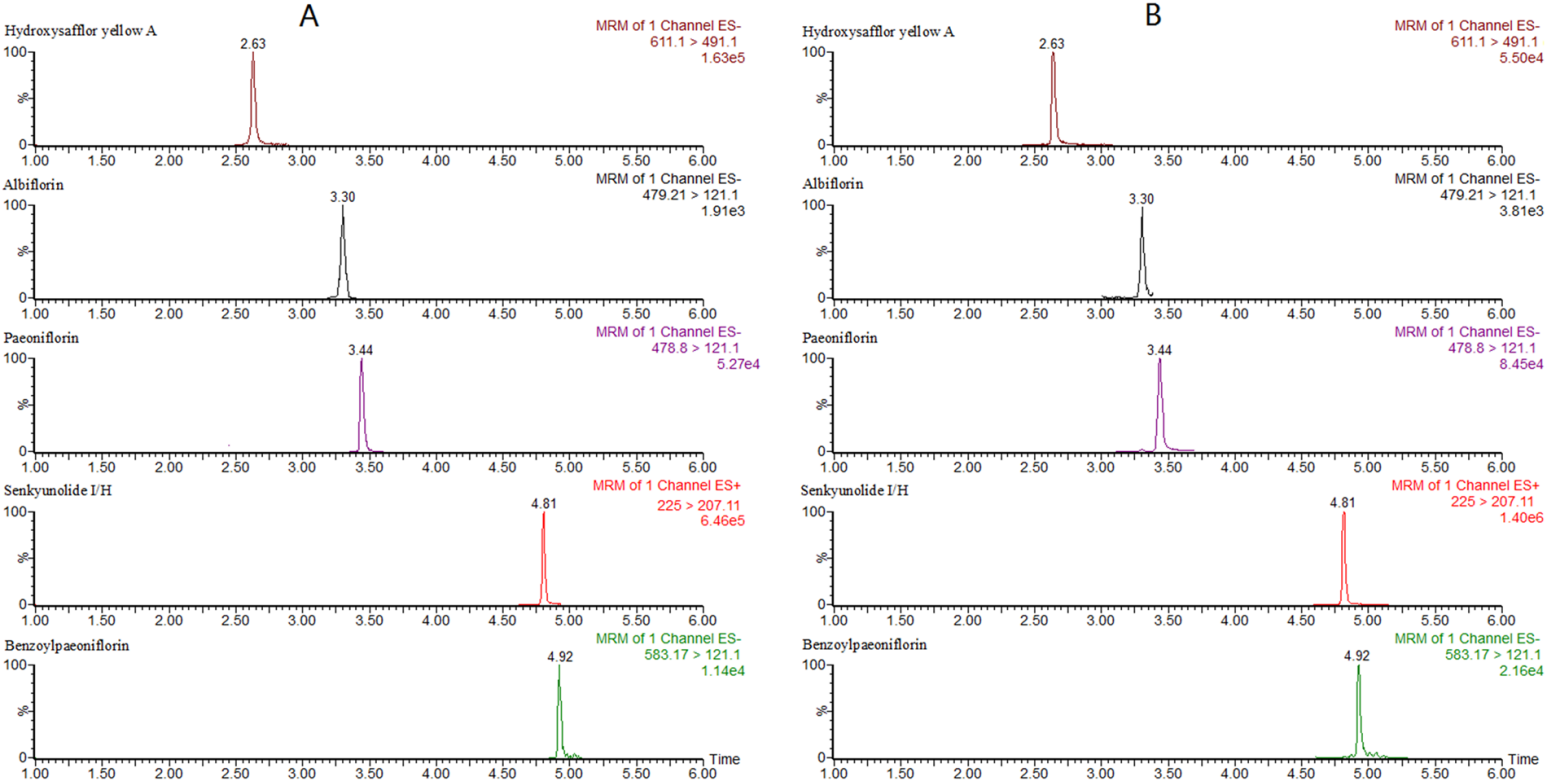
The typical chromatograms of a standard mixture of five markers (**A**) and an XBJ sample (**B**).

An UPLC-MS/MS method was developed for the routine determination of five markers in XBJ samples within 5 minutes. And the method was validated according to the above section “Method validation”. Satisfactory linearity and correlation coefficient were achieved with linear ranges. The relative standard deviations of precisions, repeatability, stability and recovery were all meeting requirements. The UPLC-MS/MS method could apply for the analysis of five marks in XBJ samples. The typical chromatograms of a standard mixture of five markers (A) and an XBJ sample (B) are shown in Fig. 5. This UPLC-MS/MS method was simpler in operation and higher in data handling efficiency for widely application.

## Methods

### Reagents and materials

HPLC grade methanol and acetonitrile for qualitative analysis were obtained from Fisher Scientific (Fair Lawn, NJ, USA). Formic acid of HPLC grade purchased from Aladdin Industrial Co., Ltd. (Shanghai, China). Ammonium acetate was MS grade and purchased from Anpel scientific instrument Corporation Ltd. (Shanghai, China). All other chemicals were of analytical reagent grade. Ultra-pure water (18.2 MΩ) was purified by Millipore system (Millipore, Shanghai, China) and all solutions were filtrated 0.22 μm pore size filters.

The reference standards of compounds A_1_-A_38_ were purchased from Chengdu Must Bio-technology Co., Ltd. (Sichuan, China). The purities of all the reference standards were over 98% and their chemical structures were illustrated in Fig. 2. Ten batches commercial patent medicines of XBJ were prepared by Tianjin Chase Sun Pharmaceutical Co., Ltd. (Tianjin, China).

### Standard solution and samples preparations

The stock standard solutions of 38 reference standards were dissolved in methanol with concentration of 1.0 mg/mL for each compound, respectively. Then, each stock solution was mixed with 50% methanol to prepare a final mixed standard solution. A series of working standard solutions were prepared by the successive dilution of the mixture of standard solutions with 50% methanol. All the solutions were stored at 4  °C before use. Ten batches of commercial preparations of XBJ were directly subjected to UHPLC-MS analysis after being filtered through a 0.22 μm syringe filter.

### Chromatographic conditions and Mass spectrometric conditions

In the quantitative analysis of 38 compounds, an UHPLC Dionex Ultimate 3000 with Q-Exactive hybrid quadrupole-orbitrap mass spectrometer system was utilized. Chromatographic peaks were separated on a Waters ACQUITY UPLC^®^ HSS C_18_ column (2.1 mm × 100 mm, 1.8 μm) at a flow rate of 0.2 mL/min with gradient acetonitrile (A) and water containing 10 mM ammonium acetate (B) as follows: 0–10 min, 5% A, 10–45 min, 5–30% A, 45–60 min, 30–100% A, and then the column was re-equilibrated at 5% A for 2 min prior to the next injection. The injection volume was 5 μL for analysis. The Q-Exactive mass spectrometer was equipped with heat electrospray ionization (HESI), an online vacuum degasser, a quaternary pumps, an autosampler, a thermostated column compartment and ultraviolet detector (UV). The optimized parameters of mass spectrometry were illustrated as below: spray voltage: + 3.5 kV or −2.8 kV; sheath gas pressure: 40 arb; Aux gas pressure: 10 arb; sweep gas pressure: 0 arb; capillary temperature: 320 °C; auxiliary gas heater temperature: 300 °C; S-lens RF level: 50 V; scan mode: (1) full MS: Resolution: 70,000; automatic gain control (AGC) target: 3.0e;^6^ maximum injection time (IT): 200 ms; scan range: 80–1200 m/z; (2) dd-MS^2^/dd-SIM: Rsolution: 17,500; AGC target: 1.0 e^5^; maximum IT: 50 ms; Loop count: 5; Isolation window: 2.0 m/z; NCE/stepped: 20, 30, 40; Dynamic exclusion: 10.0 s. Nitrogen was used for spray stabilization and as the collision gas in the C-trap. All data collected in profile mode were acquired and processed using Thermo Xcalibur 3.0 software.

In the quantitative analysis of five marks, a Waters Xevo TQD UPLC-MS/MS system (Waters Corp., Milford, MA, USA) was employed. Chromatographic peaks were separated on a Waters ACQUITY UPLC^®^ HSS C_18_ column (2.1 mm × 100 mm, 1.8 μm) at a flow rate of 0.2 mL/min with gradient acetonitrile (A) and water containing 10 mM ammonium acetate (B) as follows: 0–0.5 min, 5% A; 0.5–1.0 min, 5–20% A; 1–3.0 min, 20–30% A; 3.0–4.5 min, 30–100% A, 4.5–5.0 min, 100% A. A subsequent re-equilibration time (2 min) should be performed before next injection. The injection volume was 5 μL for analysis. The Waters Xevo TQD mass spectrometer with electrospray ion source (ESI) was used. The MS spectra were acquired in MRM mode using polarity switching. The capillary voltage was set to 3.5 kV, and the source temperature was maintained at 350 °C, nitrogen gas was used as desolvation gas 650 L/h and cone gas 50 L/h and argon gas was employed as collision gas. The most appropriate precursor-to-product ion pair, cone voltage (CV) and collision energy(CE) are listed in Table S3. All data was acquired and integrated by Masslynx V4.1 software.

## Mass spectrometric conditions

### Statistical data analysis

The fingerprinting was performed on different XBJ samples by SIEVE 2.0 software (Thermo Scientific, San Jose, USA), which was used for evaluating the similarities between different samples. The similarity was evaluated with the correlation coefficients, and the calculation of correlation coefficients was mainly based on the peak area and retention time. The base peak intensity chromatographic data obtained from the positive or negative ion UHPLC-Q-Orbitrap HRMS analyses were imported in the form of Xcalibur raw files into the SIEVE software. With SIEVE software, the chromatogram can be normalized, and the identical peaks in each chromatogram can be matched in automatic or manual mode. All the batches of XBJ samples were used to construct fingerprinting. Subsequently, the correlation coefficients of all introduced chromatograms relative to that of reference chromatogram would be calculated. In a word, the software made the analysis method accurate and rapid.

Principal component analysis (PCA) involves a mathematical procedure that transforms a number of possibly correlated variables into a smaller number of uncorrelated variables called principal components. This transformation is defined in such a way that the first principal component has as high a variance as possible or accounts for as much of the variability in the data as possible^27^. PCA is an unsupervised pattern recognition technique, which is a data visualization method useful for a rapid means of visualizing similarities or differences within multivariate data^28^. PCA makes it possible to represent objects or variables on a graph, with different objectives to study the proximity of objects in order to differentiate them and to detect atypical objects, and also to analyze the position of objects in varied representations. Thus, we could probably speculate the chemical components causing quality differences in different batches. The PCA was performed on different XBJ samples by SIMCA 14.0 software (Umetrics, Sweden).

## Electronic supplementary material

Supplementary information
