# Supplementary material for: Chemical profiling and quantification of XueBiJing injection, a systematic quality control strategy using UHPLC-Q Exactive hybrid quadrupole-orbitrap high-resolution mass spectrometry
Source: Sci Rep. 2017 Dec 5;7:16921. doi: 10.1038/s41598-017-17170-y (PMC5717239; doi:10.1038/s41598-017-17170-y)
Supplement: Supplementary file 1 — Supplementary information [file 41598_2017_17170_MOESM1_ESM.doc]

**Title Page**

**Title:**

Chemical profiling and quantification of XueBiJing injection, a systematic quality control strategy using UHPLC-Q Exactive hybrid quadrupole-orbitrap high-resolution mass spectrometry

**Authors:**

Zhi Sun, Lihua Zuo*, Tongwen Sun, Jinfa Tang, Daling Ding, Lin Zhou, Jian Kang, Xiaojian Zhang*

**Table S1** Summary of calibration parameters, LOD, LOQ, precision, repeatability and stability for the 38 bioactive compounds in XBJ.

| Compounds | Linear regression | | | LOD | LOQ | Precision RSD (%) | | Repeatability RSD (%) | | Stability |
| --- | --- | --- | --- | --- | --- | --- | --- | --- | --- | --- |
|  | Linear range (ng/ml) | Regression equation | *R^2^* | (ng/ml) | (ng/ml) | Intra-day | Inter-day | Intra-day | Inter-day | RSD (%) |
| A_1_ | 1000-10000 | *Y* = 9.05×10^7^ *X* + 1.32×10^7^ | 0.9994 | 0.18 | 0.59 | 2.09 | 2.37 | 2.56 | 1.92 | 1.53 |
| A_2_ | 2000-20000 | *Y* = 3.74×10^6^ *X* - 4.10×10^5^ | 0.9998 | 3.40 | 11.32 | 1.81 | 1.05 | 1.90 | 2.11 | 1.32 |
| A_3_ | 20-200 | *Y* = 7.85×10^8^ *X +* 5.58×10^6^ | 0.9994 | 0.04 | 0.13 | 1.04 | 1.67 | 2.96 | 1.43 | 2.05 |
| A_4_ | 120-1200 | *Y* = 6.32×10^6^ *X* - 1.00×10^4^ | 0.9994 | 0.31 | 1.03 | 1.88 | 2.48 | 1.80 | 2.56 | 1.56 |
| A_5_ | 1500-15000 | *Y* = 6.28×10^7^ *X* + 5.64×10^6^ | 0.9992 | 0.21 | 0.68 | 1.81 | 2.84 | 1.12 | 1.19 | 2.65 |
| A_6_ | 1250-12500 | *Y* = 2.19×10^7^ *X* - 6.52×10^6^ | 0.9998 | 0.63 | 2.11 | 2.43 | 1.08 | 2.34 | 2.77 | 1.18 |
| A_7_ | 1000-10000 | *Y* = 2.08×10^8^ *X* + 1.43×10^8^ | 0.9994 | 0.39 | 1.29 | 1.79 | 1.13 | 1.58 | 1.46 | 2.80 |
| A_8_ | 0.07-0.7 | *Y* = 1.89×10^8^ *X* + 4.65×10^4^ | 0.9996 | 0.05 | 0.16 | 2.82 | 2.71 | 0.99 | 1.10 | 2.17 |
| A_9_ | 1200~12000 | *Y* = 4.08×10^7^ *X* - 1.31×10^6^ | 0.9996 | 0.66 | 2.22 | 1.70 | 1.25 | 2.09 | 1.83 | 1.91 |
| A_10_ | 700-7000 | *Y* = 4.07×10^7^ *X* - 3.73×10^6^ | 0.9992 | 0.45 | 1.49 | 2.26 | 2.50 | 2.30 | 2.33 | 2.72 |
| A_11_ | 1000-10000 | *Y* = 1.10×10^8^ *X* +1.58×10^7^ | 0.9992 | 0.97 | 3.23 | 2.07 | 2.79 | 2.05 | 1.72 | 2.51 |
| A_12_ | 7500-75000 | *Y* = 2.84×10^7^ *X* +5.52×10^6^ | 0.9994 | 1.59 | 5.30 | 1.98 | 1.19 | 1.54 | 2.19 | 2.42 |
| A_13_ | 100000-1000000 | *Y* = 1.24×10^7^ *X* - 4.23×10^8^ | 0.9996 | 3.73 | 12.42 | 1.78 | 1.99 | 1.81 | 1.01 | 2.15 |
| A_14_ | 3000-30000 | *Y* = 3.72×10^5^ *X* -5.95×10^5^ | 0.9990 | 5.19 | 17.31 | 2.31 | 2.99 | 2.46 | 2.61 | 2.67 |
| A_15_ | 300000-3000000 | *Y* = 8.11×10^5^ *X* - 9.20×10^7^ | 0.9988 | 7.41 | 24.71 | 2.77 | 1.23 | 1.41 | 1.12 | 1.63 |
| A_16_ | 20000-200000 | *Y* = 4.69×10^7^ *X* +8.00×10^8^ | 0.9992 | 1.00 | 3.32 | 2.02 | 2.11 | 2.05 | 2.17 | 2.57 |
| A_17_ | 550-5500 | *Y* = 1.63×10^7^ *X* -1.93×10^5^ | 0.9992 | 1.28 | 4.27 | 1.36 | 2.78 | 1.81 | 2.09 | 2.81 |
| A_18_ | 350-3500 | *Y* = 6.55×10^7^ *X* -2.07×10^6^ | 0.9996 | 0.18 | 0.60 | 1.11 | 2.02 | 2.99 | 1.90 | 1.11 |
| A_19_ | 160-1600 | *Y* = 3.44×10^7^ *X* - 1.06×10^6^ | 0.9994 | 0.76 | 2.54 | 1.12 | 2.84 | 2.19 | 2.98 | 1.92 |
| A_20_ | 200-2000 | *Y* = 5.95×10^6^ *X+* 3.78×10^5^ | 0.9994 | 7.64 | 25.48 | 1.12 | 1.90 | 2.09 | 2.98 | 1.64 |
| A_21_ | 15000-150000 | *Y* = 6.67×10^6^ *X* - 2.52×10^7^ | 0.9992 | 3.70 | 12.35 | 2.01 | 2.57 | 1.98 | 1.87 | 1.46 |
| A_22_ | 1800-18000 | *Y* = 8.93×10^7^ *X +* 3.02×10^6^ | 0.9992 | 0.14 | 0.47 | 2.06 | 1.31 | 2.61 | 1.69 | 2.69 |
| A_23_ | 1000-10000 | Y = 2.68×10^5^ X - 1.62×10^5^ | 0.9996 | 10.59 | 35.29 | 1.78 | 2.89 | 2.54 | 2.69 | 2.86 |
| A_24_ | 700-7000 | *Y* = 3.73×10^7^ *X* - 3.79×10^6^ | 0.9996 | 0.35 | 1.18 | 1.18 | 2.06 | 1.25 | 1.80 | 1.61 |
| A_25_ | 15-150 | *Y* = 2.51×10^8^ *X* - 9.83×10^6^ | 0.9992 | 0.17 | 0.55 | 1.04 | 1.13 | 2.69 | 0.92 | 2.25 |
| A_26_ | 8-80 | *Y* = 6.25×10^8^ *X* - 4.57×10^5^ | 0.9992 | 0.04 | 0.13 | 2.48 | 1.83 | 3.00 | 2.90 | 2.21 |
| A_27_ | 80-800 | *Y* = 1.97×10^8^ *X* - 1.57×10^6^ | 0.9994 | 0.12 | 0.41 | 1.82 | 2.97 | 2.48 | 2.54 | 1.94 |
| A_28_ | 5-50 | *Y* = 2.07×10^8^*X -*5.33×10^4^ | 0.9992 | 0.27 | 0.89 | 1.49 | 1.93 | 1.82 | 2.54 | 2.39 |
| A_29_ | 120-1200 | *Y* = 1.93×10^8^ *X* - 1.81×10^6^ | 0.9994 | 3.23 | 10.78 | 0.95 | 1.83 | 2.11 | 2.07 | 1.38 |
| A_30_ | 7500-75000 | *Y* = 1.32×10^6^ *X +* 5.06×10^5^ | 0.9994 | 35.77 | 119.22 | 2.27 | 1.91 | 1.13 | 1.22 | 2.09 |
| A_31_ | 40-400 | *Y* = 5.88×10^7^ *X* + 9.85×10^5^ | 0.9996 | 1.32 | 4.40 | 2.78 | 1.21 | 2.99 | 1.22 | 2.83 |
| A_32_ | 250-2500 | *Y* = 2.30×10^8^ *X* - 2.34×10^7^ | 0.9994 | 0.06 | 0.20 | 1.82 | 2.43 | 1.54 | 2.37 | 1.13 |
| A_33_ | 100-1000 | *Y* = 3.16×10^8^ *X* + 5.70×10^6^ | 0.9994 | 0.02 | 0.07 | 1.40 | 1.74 | 2.88 | 1.88 | 2.58 |
| A_34_ | 13-130 | *Y* = 5.79×10^8^ *X* + 5.68×10^6^ | 0.9992 | 0.03 | 0.08 | 1.89 | 2.64 | 2.37 | 1.54 | 2.51 |
| A_35_ | 2.5-25 | *Y* = 4.40×10^7^ *X* + 1.08×10^4^ | 0.9992 | 0.23 | 0.76 | 2.28 | 2.20 | 1.04 | 2.54 | 2.73 |
| A_36_ | 100-1000 | *Y* = 1.12×10^9^ *X* + 8.97×10^6^ | 0.9992 | 0.01 | 0.03 | 2.88 | 1.51 | 2.82 | 1.44 | 2.77 |
| A_37_ | 0.3-3 | *Y* = 2.67×10^8^ *X* - 9.85×10^3^ | 0.9994 | 0.08 | 0.28 | 2.21 | 2.89 | 1.34 | 2.91 | 1.93 |
| A_38_ | 11-110 | *Y* = 5.54×10^6^ *X* - 2.44×10^3^ | 0.9994 | 1.91 | 6.36 | 1.54 | 1.59 | 2.14 | 2.27 | 1.86 |

**Table S2**.The results of recoveries of 38 compounds (n = 3)

| Compounds | Spiked (μg) | | | Found (μg) | | | Mean recovery (%) | RSD  (%) |
| --- | --- | --- | --- | --- | --- | --- | --- | --- |
|  | low | medium | high | low | medium | high |  |  |
| A_1_ | 2.881 | 3.601 | 4.322 | 6.476 | 7.202 | 7.934 | 100.0 | 2.24 |
| A_2_ | 5.815 | 7.268 | 8.722 | 13.172 | 14.489 | 16.067 | 100.6 | 2.69 |
| A_3_ | 0.034 | 0.043 | 0.052 | 0.077 | 0.085 | 0.095 | 99.9 | 2.21 |
| A_4_ | 0.322 | 0.403 | 0.483 | 0.729 | 0.808 | 0.883 | 100.4 | 1.84 |
| A_5_ | 1.204 | 1.505 | 1.806 | 2.703 | 3.023 | 3.322 | 100.3 | 1.61 |
| A_6_ | 1.788 | 2.235 | 2.682 | 3.998 | 4.461 | 4.866 | 98.7 | 1.93 |
| A_7_ | 1.990 | 2.487 | 2.985 | 4.449 | 4.939 | 5.434 | 98.6 | 1.50 |
| A_8_ | 0.002 | 0.002 | 0.002 | 0.004 | 0.004 | 0.004 | 98.6 | 2.04 |
| A_9_ | 1.734 | 2.167 | 2.600 | 3.884 | 4.342 | 4.755 | 99.6 | 2.02 |
| A_10_ | 1.359 | 1.699 | 2.039 | 3.069 | 3.400 | 3.752 | 100.5 | 2.37 |
| A_11_ | 1.893 | 2.367 | 2.840 | 4.281 | 4.737 | 5.245 | 100.9 | 2.07 |
| A_12_ | 14.454 | 18.068 | 21.682 | 32.453 | 36.060 | 39.810 | 99.8 | 2.27 |
| A_13_ | 234.954 | 293.693 | 352.431 | 532.751 | 586.105 | 654.818 | 101.3 | 1.76 |
| A_14_ | 6.633 | 8.291 | 9.949 | 15.105 | 16.372 | 18.385 | 100.6 | 2.62 |
| A_15_ | 357.406 | 446.757 | 536.109 | 807.474 | 889.467 | 981.317 | 99.9 | 1.94 |
| A_16_ | 12.397 | 15.496 | 18.596 | 27.900 | 30.801 | 34.246 | 99.9 | 2.98 |
| A_17_ | 1.604 | 2.005 | 2.406 | 3.594 | 3.973 | 4.452 | 99.6 | 2.37 |
| A_18_ | 0.162 | 0.202 | 0.243 | 0.363 | 0.401 | 0.440 | 98.6 | 1.87 |
| A_19_ | 0.489 | 0.612 | 0.734 | 1.107 | 1.231 | 1.346 | 100.8 | 1.72 |
| A_20_ | 0.446 | 0.557 | 0.669 | 1.006 | 1.114 | 1.220 | 99.8 | 2.27 |
| A_21_ | 34.113 | 42.641 | 51.169 | 77.071 | 85.879 | 94.326 | 101.1 | 1.86 |
| A_22_ | 2.384 | 2.980 | 3.576 | 5.311 | 5.962 | 6.611 | 99.8 | 2.34 |
| A_23_ | 0.661 | 0.827 | 0.992 | 1.488 | 1.654 | 1.798 | 99.3 | 2.76 |
| A_24_ | 0.904 | 1.131 | 1.357 | 2.055 | 2.270 | 2.479 | 100.8 | 2.27 |
| A_25_ | 0.049 | 0.062 | 0.074 | 0.112 | 0.124 | 0.136 | 100.7 | 1.45 |
| A_26_ | 0.016 | 0.020 | 0.023 | 0.035 | 0.039 | 0.043 | 100.5 | 2.74 |
| A_27_ | 0.140 | 0.175 | 0.210 | 0.312 | 0.349 | 0.390 | 99.9 | 2.75 |
| A_28_ | 0.010 | 0.012 | 0.015 | 0.022 | 0.025 | 0.027 | 100.5 | 1.37 |
| A_29_ | 0.180 | 0.226 | 0.271 | 0.405 | 0.452 | 0.502 | 100.5 | 2.23 |
| A_30_ | 12.010 | 15.013 | 18.015 | 27.049 | 29.860 | 32.875 | 99.4 | 2.54 |
| A_31_ | 0.082 | 0.103 | 0.124 | 0.185 | 0.208 | 0.225 | 100.4 | 2.34 |
| A_32_ | 0.147 | 0.184 | 0.221 | 0.329 | 0.373 | 0.403 | 99.7 | 2.63 |
| A_33_ | 0.078 | 0.098 | 0.117 | 0.174 | 0.196 | 0.214 | 98.7 | 2.24 |
| A_34_ | 0.007 | 0.009 | 0.011 | 0.017 | 0.019 | 0.020 | 100.3 | 2.24 |
| A_35_ | 0.008 | 0.010 | 0.012 | 0.018 | 0.020 | 0.022 | 98.5 | 2.03 |
| A_36_ | 0.189 | 0.236 | 0.284 | 0.427 | 0.476 | 0.524 | 101.1 | 1.94 |
| A_37_ | 0.00019 | 0.00023 | 0.00028 | 0.00041 | 0.00046 | 0.00051 | 99.3 | 2.71 |
| A_38_ | 0.034 | 0.042 | 0.051 | 0.076 | 0.084 | 0.093 | 100.3 | 2.09 |

**Table S3** Molecular weight (MW), ion mode, MRM transitions, cone voltage(CV) collision energy(CE) and retention time(RT) of five marks in Xuebijing.

| Analytes | MW | ion mode | Transitions | CV (V) | CE (eV) | RT |
| --- | --- | --- | --- | --- | --- | --- |
| Hydroxysafflor yellow A | 611.1 | negtive | 611.1→491.1 | 64 | 28 | 2.63 |
| Albiflorin | 479.2 | negtive | 479.2→121.1 | 52 | 22 | 3.30 |
| Paeoniflorin | 478.8 | negtive | 478.8→121.1 | 28 | 14 | 3.44 |
| Senkyunolide I/H | 225.0 | positive | 225.0→207.1 | 18 | 10 | 4.81 |
| Benzoylpaeoniflorin | 583.1 | negtive | 583.1→121.1 | 54 | 24 | 4.92 |
